# Trivalent mosaic or consensus HIV immunogens prime humoral and broader cellular immune responses in adults

**DOI:** 10.1172/JCI163338

**Published:** 2023-02-15

**Authors:** Kristen W. Cohen, Andrew Fiore-Gartland, Stephen R. Walsh, Karina Yusim, Nicole Frahm, Marnie L. Elizaga, Janine Maenza, Hyman Scott, Kenneth H. Mayer, Paul A. Goepfert, Srilatha Edupuganti, Giuseppe Pantaleo, Julia Hutter, Daryl E. Morris, Stephen C. De Rosa, Daniel E. Geraghty, Merlin L. Robb, Nelson L. Michael, Will Fischer, Elena E. Giorgi, Harmandeep Malhi, Michael N. Pensiero, Guido Ferrari, Georgia D. Tomaras, David C. Montefiori, Peter B. Gilbert, M. Juliana McElrath, Barton F. Haynes, Bette T. Korber, Lindsey R. Baden, the NIAID HVTN 106 Study Group

**Affiliations:** 1 Vaccine and Infectious Disease Division, Fred Hutchinson Cancer Center, Seattle, Washington, USA.; 2 Division of Infectious Diseases, Brigham and Women’s Hospital, Boston, Massachusetts, USA.; 3 Harvard Medical School, Boston, Massachusetts, USA.; 4 Center for Virology and Vaccine Research, Beth Israel Deaconess Medical Center, Boston, Massachusetts, USA.; 5 Theoretical Biology and Biophysics Group, Los Alamos National Laboratory, and New Mexico Consortium, Los Alamos, New Mexico, USA.; 6 Department of Global Health, University of Washington, Seattle, Washington, USA.; 7 San Francisco Department of Public Health, San Francisco, California, USA.; 8 The Fenway Institute, Fenway Health, Boston, Massachusetts, USA.; 9 University of Alabama at Birmingham, Birmingham, Alabama, USA.; 10 Emory University, Atlanta, Georgia, USA.; 11 Centre Hospitalier Universitaire Vaudois, Lausanne, Switzerland.; 12 Division of AIDS, National Institute of Allergy and Infectious Diseases, Bethesda, Maryland, USA.; 13 Henry M. Jackson Foundation for the Advancement of Military Medicine, Bethesda, Maryland, USA.; 14 Walter Reed Army Institute of Research, Silver Spring, Maryland, USA.; 15 Duke Human Vaccine Institute and; 16 Department of Surgery, Duke University, Durham, North Carolina, USA.; 17 The NIAID HVTN 106 Study Group is detailed in Supplemental Acknowledgments.

**Keywords:** AIDS/HIV, Vaccines, AIDS vaccine, Cellular immune response

## Abstract

**BACKGROUND:**

Mosaic and consensus HIV-1 immunogens provide two distinct approaches to elicit greater breadth of coverage against globally circulating HIV-1 and have shown improved immunologic breadth in nonhuman primate models.

**METHODS:**

This double-blind randomized trial enrolled 105 healthy HIV-uninfected adults who received 3 doses of either a trivalent global mosaic, a group M consensus (CON-S), or a natural clade B (Nat-B) gp160 *env* DNA vaccine followed by 2 doses of a heterologous modified vaccinia Ankara–vectored HIV-1 vaccine or placebo. We performed prespecified blinded immunogenicity analyses at day 70 and day 238 after the first immunization. T cell responses to vaccine antigens and 5 heterologous Env variants were fully mapped.

**RESULTS:**

Env-specific CD4^+^ T cell responses were induced in 71% of the mosaic vaccine recipients versus 48% of the CON-S recipients and 48% of the natural Env recipients. The mean number of T cell epitopes recognized was 2.5 (95% CI, 1.2–4.2) for mosaic recipients, 1.6 (95% CI, 0.82–2.6) for CON-S recipients, and 1.1 (95% CI, 0.62–1.71) for Nat-B recipients. Mean breadth was significantly greater in the mosaic group than in the Nat-B group using overall (*P* = 0.014), prime-matched (*P* = 0.002), heterologous (*P* = 0.046), and boost-matched (*P* = 0.009) measures. Overall T cell breadth was largely due to Env-specific CD4^+^ T cell responses.

**CONCLUSION:**

Priming with a mosaic antigen significantly increased the number of epitopes recognized by Env-specific T cells and enabled more, albeit still limited, cross-recognition of heterologous variants. Mosaic and consensus immunogens are promising approaches to address global diversity of HIV-1.

**TRIAL REGISTRATION:**

ClinicalTrials.gov NCT02296541.

**FUNDING:**

US NIH grants UM1 AI068614, UM1 AI068635, UM1 AI068618, UM1 AI069412, UL1 RR025758, P30 AI064518, UM1 AI100645, and UM1 AI144371, and Bill & Melinda Gates Foundation grant OPP52282.

## Introduction

The tremendous global genetic diversity of HIV-1 poses a formidable challenge in the development of a globally effective HIV-1 vaccine (1, 2). Several vaccine concepts have completed efficacy trials (3–8), but only a single study (RV144) has shown significant reduction in HIV-1 acquisition following vaccination (7). Immune-correlates analyses of RV144 suggested that both non-neutralizing antibodies (9–11) and polyfunctional vaccine-specific CD4^+^ T cells (12) were associated with reduced risk of HIV-1 infection in the trial. A subsequent analysis of a DNA-prime, recombinant adenovirus–vectored vaccine trial (HVTN 505) showed that envelope-specific (Env-specific) CD8^+^ T cells with high-magnitude polyfunctionality were associated with reduced risk of infection (13), despite a lack of vaccine-induced protection overall (6). In a trial of a different recombinant adenovirus 5 (Ad5) vaccine there was no association of T cell responses with HIV-1 infection, but analyses found a correlation between the number of Gag T cell epitopes recognized and lower viral load among those who became infected (14, 15). This was consistent with the role of CD8^+^ T cells in the context of natural infection, for which control of viremia has long been correlated with breadth of CD8^+^ T cell recognition (16–18), particularly breadth of epitopes in more conserved regions of the proteome (19–22). Also, vaccine-elicited CD8^+^ T cell responses have been found to exert selective pressure on infecting viruses (23). Despite these analyses linking T cell responses to benefits in vaccine trials, protection from infection was modest in RV144, and other regimens did not provide efficacy against infection nor improve control over postinfection viremia, perhaps because an insufficient number of epitopes (14) were recognized or because highly variable regions of the viral genome (24) were targeted.

In nonhuman primate studies, the number of vaccine-elicited T cells has been associated with reduced peak viremia and set point (2, 25, 26) as well as improved survival (27, 28). Moreover, higher levels of vaccine-elicited T cell responses have been found to be a correlate of protection from heterologous simian-human immunodeficiency virus (SHIV) challenge (29, 30). T cell responses to Gag delivered using a serial multivector approach were shown to enhance vaccine protection from challenge by complementing non-neutralizing antibody responses and enabling more durable protection with lower neutralizing antibody titers (29, 31). These observations suggest that HIV-specific T cell responses can contribute to vaccine-mediated viral prevention and control. Thus, we hypothesize that we can improve the potential for viral control by increasing the cross-reactive potential between vaccine-elicited responses and natural variants encountered.

To counter global HIV-1 diversity, the ideal vaccine would elicit T cell responses against multiple distinct epitopes (i.e., breadth) with the ability to cross-react with diverse variants within targeted epitope regions (i.e., depth). Several strategies to increase breadth and depth have been proposed, including computationally derived mosaic (32–35), consensus (36), and conserved region antigens (21). Mosaic immunogens are in silico–derived recombinant protein sequences optimized for maximal inclusion of potential T cell epitopes based on the diversity in a target population and used in combinations for complementarity. They capture the most common circulating forms of variable epitopes and are designed to allow natural expression and antigen processing and presentation. Studies of mosaic HIV-1 immunogens in rhesus macaques have shown that they can increase the breadth and depth of cellular immune responses compared with either consensus or natural sequences (37, 38). Consensus sequences, in contrast, simply represent the most common amino acid (aa) in each position among an alignment of available viral sequences (39); the group M consensus (CON-S) sequence we used here is a consensus of the major HIV-1 subtype consensus sequences (36). By presenting the most common forms of circulating epitopes, mosaic and consensus immunogen approaches could elicit responses that enhance the cross-reactive potential of vaccine-elicited responses against diverse circulating viruses, enabling better control of viremia and limiting in vivo evolution of variant “escape” viruses. Additionally, mosaic immunogens could improve priming of CD4^+^ T cells such that they are better able to cross-recognize a heterologous boost immunogen, thereby improving antibody responses in heterologous prime-boost vaccine strategies. Since many HIV vaccine strategies in development are focused on induction and maturation of neutralizing antibody responses through sequential immunizations (40), it is critical to design immunogens that can serve as “universal” primes to provide T cell help for heterologous sequential boosting strategies.

In this study, we report the first-in-human safety and immunogenicity evaluation of 3 different DNA-prime HIV-1 vaccines: a natural clade B (Nat-B) *env* vaccine, a CON-S *env* vaccine, and a trivalent mosaic *env* combination vaccine. As antibody and CD4^+^ T cell responses were found to be among the correlates of the RV144 efficacy trial (9), we chose a DNA-prime and poxvirus-boost delivery system for this vaccine regimen. The boost carried a circulating recombinant form 01 (CRF01) natural variant Env antigen that closely resembled the RV144 vaccine antigen. We evaluated the capacity of these novel DNA immunogens to induce broad, cross-clade immune responses that could prime the response by a heterologous immunogen, delivered by a modified vaccinia Ankara (MVA) vector.

## Results

### Participant characteristics and demographics.

**Figure 1:**
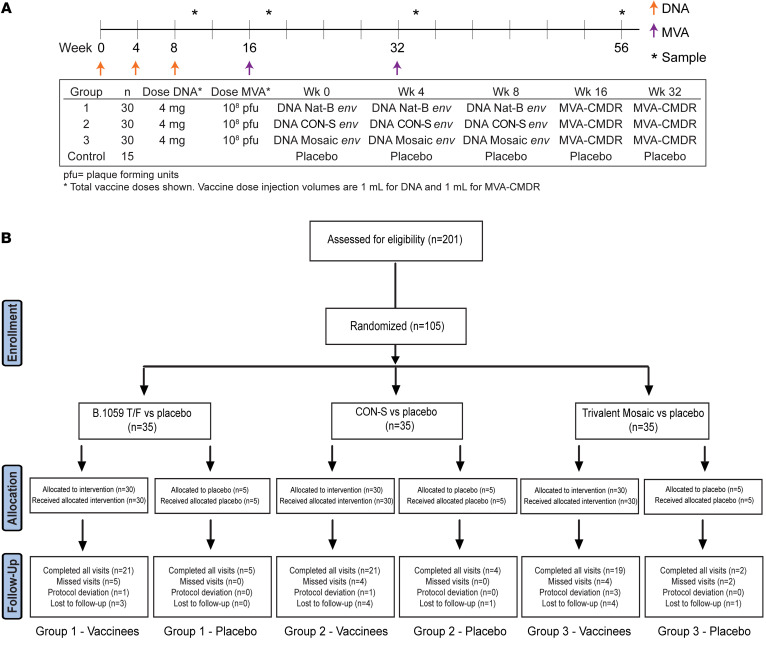
HVTN 106 study schema and CONSORT diagram. The study schema (**A**) and screening, enrollment, and retention (**B**) for the HVTN 106 study. Healthy, HIV-negative individuals were screened at 7 sites in the United States and Switzerland. Participants meeting the eligibility and enrollment criteria were randomized to receive placebo or 1 of 3 vaccine regimens. Participants were followed for safety and immunogenicity with regular visits through 14 months.

Beginning on December 16, 2014, 105 participants were enrolled through September 8, 2015, at 7 sites in the United States and Switzerland (Figure 1); 61 participants (58%) were male, the median age was 30 years (range 18–50 years), and 66% were White (Supplemental Table 1; supplemental material available online with this article; https://doi.org/10.1172/JCI163338DS1). Overall retention was 93% (1,262 of 1,364 planned visits completed), and 491 of 525 (94%) of the planned vaccinations were given.

### Safety and tolerability.

The mosaic, CON-S, and subtype B strain (Nat-B) DNA and MVA-CMDR vaccines were safe and generally well tolerated (Supplemental Figure 1). Local reactogenicity symptoms (including pain, tenderness, and/or induration) were more commonly reported by participants randomized to vaccine than placebo. Systemic reactogenicity symptoms did not differ among the groups. No serious adverse events related to the vaccine occurred. At least 1 adverse event (AE) occurred in 91 participants (87%), no laboratory abnormalities greater than grade 1 (mild) were observed, and no related cardiac events (41) were noted. All of the grade 2 (moderate) or higher AEs were deemed not related to study vaccination by site investigators.

### HIV-1 Env-specific polyfunctional antibody responses.

**Figure 2:**
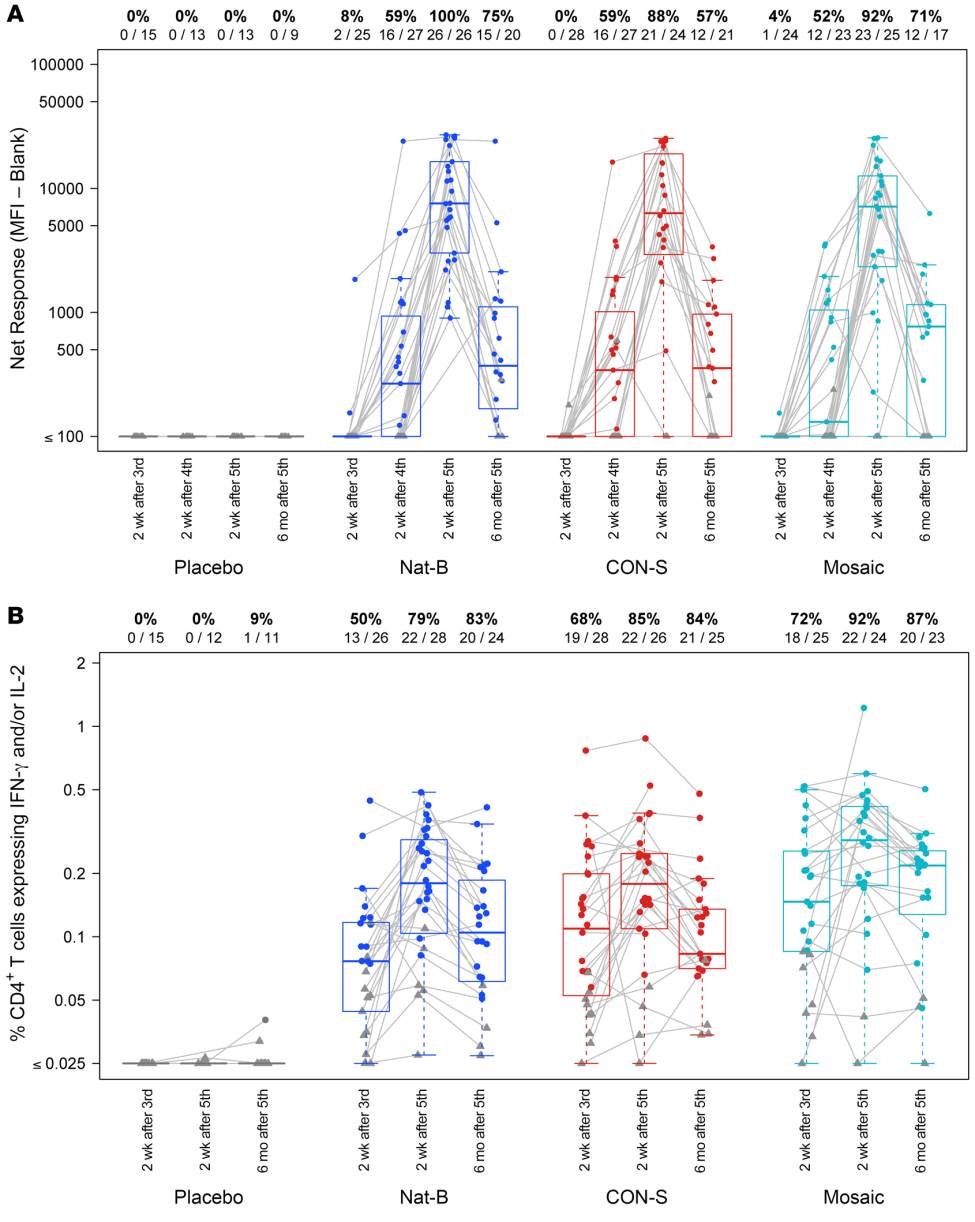
HIV-1 Env-specific IgG antibody and CD4^+^ T cell responses. (**A**) IgG binding antibody responses to HIV-1 gp140 (using AE.01.CON.03, the closest match to AE.CM235 in the MVA boost) measured by the binding antibody multiplex assay (BAMA) at 1:50 dilution from samples collected at 2 weeks after third DNA (day 70), 2 weeks after first MVA (day 126), 2 weeks after second MVA (day 238), and 6 months after second MVA (day 425, last visit). (**B**) Frequency of HIV-1 Env-specific CD4^+^ T cells was measured by intracellular cytokine staining (ICS) at 2 weeks after third DNA (day 70), 2 weeks after second MVA (day 238), and 6 months after second MVA (day 425, last visit) from cryopreserved PBMCs. Responding cells expressed either IFN-γ or IL-2 in response to 1 of 3 PTE-global 15-mer peptide pools; the summed frequency across these pools is displayed. For **A** and **B**, overlaid box plots show the median and interquartile range (IQR) among responders (colored circles) and nonresponders (gray triangles) in each treatment group (see Methods for BAMA and ICS response call details); whiskers extend to the most extreme data points that are no more than 1.5 times the IQR. Lines connect samples from the same individual. Number and percentage of positive responses are indicated along the top of each panel for each group and time point.

Two weeks after the third DNA vaccination, there were weak and sparse binding antibody responses detected against the vaccine-matched antigens with no significant differences between the groups (Figure 2A). However, these binding antibodies were rapidly boosted by a single heterologous MVA-CMDR boost and reached peak titers after the second MVA-CMDR boost (Figure 2A). At peak immunogenicity, these binding antibodies were functional and mediated cross-reactive antibody-dependent cellular cytotoxicity (ADCC) in vitro against HIV-1 infectious molecular clones expressing Envs matched to the vaccine boost, CRF01_AE CM235 (Supplemental Figure 2A). All vaccine groups had similar response rates and magnitudes of ADCC-mediated granzyme B uptake by A244, CM235, and MN gp120-coated target cells, with the highest ADCC responses being detected against the subtype B (MN) strain (Supplemental Figure 2B). Together these data show that the mosaic, consensus, and subtype B DNA immunogens consistently primed Env-specific binding antibody responses. As anticipated, given the delivery and forms of the Env antigen, limited HIV-specific neutralizing Ab responses were detected and only against tier 1 viruses (Supplemental Figure 3).

### Priming with the mosaic DNA induced higher frequencies of HIV-1 Env-specific polyfunctional CD4^+^ T cells.

Following the third DNA vaccination, Env-specific CD4^+^ T cell responses were detected in a significantly higher proportion of the participants who received the mosaic vaccine compared with those who received the Nat-B vaccine (72% vs. 50%; *P* = 0.0077) as measured by intracellular cytokine staining (ICS) assay. Env-specific CD4^+^ T cell responses in the mosaic group were only marginally higher than those in the CON-S group (62%) and not significantly different after the third vaccination (Figure 2B). Increased polyfunctional Env-specific CD4^+^ T cells (as indicated by the polyfunctionality score from the COMPASS analysis) were detected in the mosaic- and CON-S–vaccinated groups compared with the Nat-B–primed group after the third vaccination (*P* = 0.005; Supplemental Figure 4). Env-specific CD8^+^ T cell responses were detected at low but similar frequencies between groups post-DNA. While T cell responses were not evaluated after the first MVA boost, all active vaccine groups had significant increases in Env-specific CD4^+^ and CD8^+^ T cell response magnitudes and response rates after the second MVA-CMDR boost (after fifth vaccination; Figure 2B and Supplemental Figure 5). However, there were no significant differences in overall magnitudes, response rates, or polyfunctionality scores of Env-specific CD4^+^ or CD8^+^ T cell responses between vaccine groups after the second MVA-CMDR boost. The cytokine expression profile of the Env-specific CD4^+^ and CD8^+^ T cell subsets was relatively stable from the time point after the repeated DNA vaccination (after third vaccination) to the time point after boosting with the heterologous MVA (after fifth vaccination). The polyfunctional Env-specific CD4^+^ T cell responses did not include detectable expression of IL-4, but instead were characterized by expression of IFN-γ, IL-2, TNF-α, and CD40L with or without granzyme B (Supplemental Figure 6A). In contrast, the polyfunctional Env-specific CD8^+^ T cell subsets were characterized by expression of IFN-γ, TNF-α, and granzyme B, with or without IL-2 (Supplemental Figure 6B).

### Mosaic DNA primed broader HIV-1 Env-specific T cell responses.

The core objective of the mosaic Env concept is to elicit T cell responses that can better interact with the commonly circulating forms of the virus than natural variants by including the most common variants of potential epitopes in the vaccine. Therefore, we determined the capacity of T cell responses elicited by the vaccines tested in this study to interact directly with heterologous variants representative of natural circulating forms. An extension of this hypothesis is that mosaics should enable priming of broad T cell responses that could also provide improved boosting with heterologous vaccine immunogens. To assess vaccine-elicited cross-reactive T cells, we synthesized 15-mer peptides overlapping by 11 aa spanning the Env proteins to cover all variants comprising the 6 vaccine-matched Env sequences including the DNA-prime immunogens (Mos-1, Mos-2, Mos-3, CON-S, B.1059) and the MVA boost (01.CM235), as well as 5 heterologous circulating HIV-1 Env sequences (B.US.2011, B.ZA.2009, B.ES.2010, A1.KE.2009, and C.ZA.2009), for a total of 1,948 peptides (Supplemental Excel Files 1–3). We used PBMC samples collected 2 weeks after the second MVA-boost vaccination (fifth injection) to map peptide-level T cell responses using the IFN-γ ELISpot assay. Since CD4^+^ T cells do not always secrete high levels of IFN-γ, we first confirmed that the Env-specific CD4^+^ T cells detected in our ICS data expressed sufficient IFN-γ. Indeed, the Env-specific IFN-γ^+^ CD4^+^ T cell response rates (81.5%–85.7%) were very similar to those detected by IFN-γ and/or IL-2 expression (78.6%–91.7%).

Epitope testing was conducted in 3 stages, with subsequent testing conditioned on a positive result. For the initial stage of mapping, sequential pools of approximately 80 peptides each were tested. For each participant’s pool with a positive response, mini-pools of 8 to 15 (median 13) peptides were tested in a second stage of mapping. Generally, each mini-pool contained multiple variants of overlapping peptides covering the same epitope region. The distinct 15-mers from positive mini-pools were tested individually in the third and final stage.

**Figure 3:**
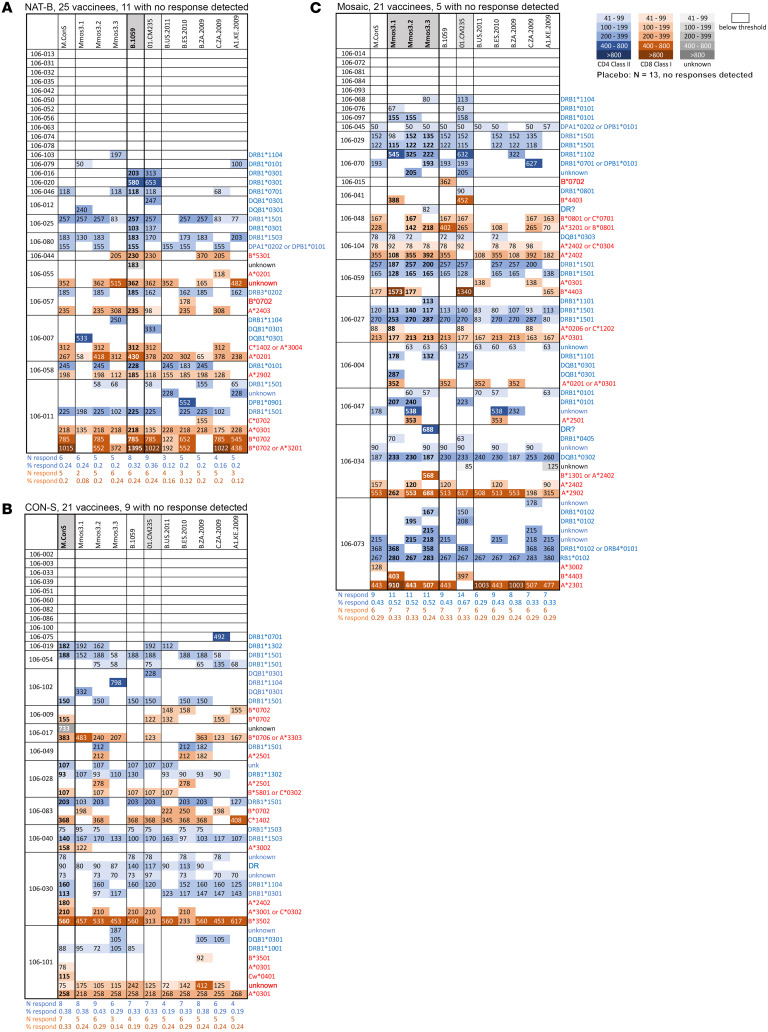
T cell ELISpot responses to vaccine-matched and heterologous HIV-1 envelope 15-mer peptides. T cell epitope mapping was conducted with PBMC samples collected 2 weeks after second MVA (day 238) using IFN-γ ELISpot. Using a response-conditional, hierarchical pooling approach, 15-mer peptides spanning envelope (11 aa overlap) were used from the 6 vaccine immunogens (NAT-B [B.1059], CON-S [M.ConS], mosaic [Mmos3.1, Mmos3.2, Mmos3.3], MVA [01.CM235]) and 5 heterologous circulating strains (B.US.2011, B.ZA.2009, B.ES.2010, A1.KE.2009, C.ZA.2009). The vaccine-matched peptides for each group are indicated by gray shading at the top of each column. Only participants with a significant T cell response by ICS were included in the epitope mapping, with each row of the tables representing an individual in the Nat-B (**A**, *n* = 25 mapped), CON-S (**B**, *n* = 21 mapped), or mosaic (**C**, *n* = 21 mapped) treatment group, and positive responses annotated with their single-peptide response magnitude (spot-forming cells per million). Determinations of the responding T cell subset were made using single-peptide ICS experiments (indicated by the color of the rectangle: blue, CD4^+^; red, CD8^+^; gray, unknown). Each row of the tables indicates a response to a single epitope across variants. Often these epitopes are variable between strains, and a complete mapping of targeted epitope variation is available in Supplemental Excel Files 1–3. Empty rows indicate that no responses were detected in a given individual. The number and percentage of individuals with at least one CD4^+^ or CD8^+^ response are indicated below each table. The restricting HLA allele for each response was determined using peptide binding prediction (NetMHCpan 4.0), a database of previously observed HIV-1 responses (Los Alamos National Laboratory), and the participant’s HLA genotype.

**Figure 4:**
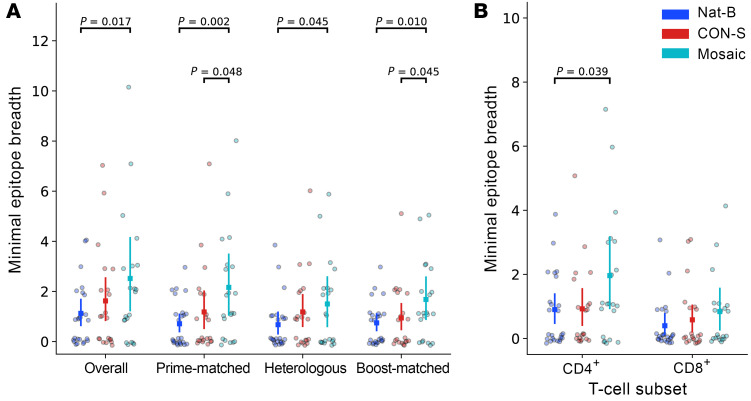
T cell response epitope breadth. The minimum number of T cell epitopes that could explain each participant’s peptide responses (i.e., epitope breadth) was determined from the participant’s ELISpot responses to HIV-1 envelope 15-mers and on the basis of the assumption that responses to 2 or more 15-mers sharing a region of ≥8 positions can be explained as a response to a single epitope (see Methods for description of the prespecified, deterministic algorithm for identifying targeted epitopes). (**A**) Breadth was estimated based on all of the peptides/responses (Overall) or on a subset of the peptides matching the DNA (Prime-matched), circulating strains (Heterologous), or the MVA immunogen (Boost-matched). Each participant is plotted (colored circle) with a treatment group mean and 95% confidence interval (non-parametric bootstrap) overlaid. Groups were compared using a permutation test on the mean (2-sided); significant differences (unadjusted *P* < 0.05) are annotated with a *P* value. (**B**) Overall epitope breadth was also analyzed separately for CD4^+^ and CD8^+^ T cell responses (determined by ICS). A small, arbitrary amount of “jitter” along the *x* axis has been added to each data point to increase visibility.

In the third stage, 835 individual 15-mer peptides were tested using samples from 51 participants for a total of 2,692 tests (plus negative and positive controls). Of these, we identified 424 peptide-specific positive responses among 42 participants (Figure 3). Many participants responded to multiple overlapping peptides, which in some cases represent responses to a single epitope. Therefore, to compute a conservative estimate of response breadth, we applied an algorithm that has been used in prior studies (42, 43) to resolve the minimum set of epitopes needed to explain each participant’s pattern of peptide responses: if 2 peptides overlapping by at least 8 aa were both positive, it was counted as a single response. Application of this algorithm indicated that at least 107 epitopes were recognized among the 15-mer peptide responses (Figure 4). Minimal epitope response breadth was calculated for each participant by counting of the number of epitopes that were identified using (a) all the peptides (overall breadth), (b) peptides derived from DNA prime–matched sequences, (c) peptides derived from the 5 heterologous natural sequences, or (d) peptides derived from the MVA boost–matched sequence (Figure 4A). Participants in the mosaic vaccine group recognized an average of 2.5 epitopes overall (95% CI, 1.2–4.2) with participants in the CON-S and Nat-B groups recognizing 1.6 (95% CI, 0.82–2.6) and 1.1 (95% CI, 0.62–1.71) epitopes on average. Mean breadth in the mosaic group was significantly greater than that in the Nat-B group using the overall (*P* = 0.017), prime-matched (*P* = 0.002), heterologous (*P* = 0.045), and boost-matched (*P* = 0.010) measures. The mosaic group also had greater mean breadth than the CON-S group by the prime-matched (*P* = 0.048) and the boost-matched (*P* = 0.045) peptides. All other group differences were not statistically significant.

To determine whether the HIV-specific T cell responses were mediated by CD4^+^ or CD8^+^ T cells, each positive peptide ELISpot response was retested with the same peptide in an ICS assay using PBMCs from the same visit (2 weeks after the final vaccination). Overall, 51% of the peptides tested were positive only for a CD4^+^ T cell response, 37% only for a CD8^+^ T cell response, 3.5% were positive for both a CD4^+^ and a CD8^+^ T cell response, and 8% could not be confirmed by ICS. For many of the peptides that did not elicit a response by ICS there was an overlapping peptide that did elicit a response. Based on the ICS responses, we determined whether each epitope detected by ELISpot (107 epitopes) could be explained by a CD4^+^ T cell response (62 epitopes), a CD8^+^ T cell response (23 epitopes), or both CD4^+^ and CD8^+^ T cell responses (14 epitopes) or whether it was indeterminate (8 epitopes). With these data we computed CD4^+^ and CD8^+^ minimal epitope breadth for each participant and compared the group means (Figure 4B). Consistent with the overall results, the CD4^+^ T cell breadth was greater in the mosaic group compared with the Nat-B (*P* = 0.039). However, there was no significant difference in the CD8^+^ T cell breadth among the vaccine groups, suggesting that the difference in overall T cell breadth was specifically driven by Env-specific CD4^+^ T cell responses.

### Variant recognition across vaccine groups.

To compare the ability of vaccinees in the different groups to elicit responses that can recognize heterologous variants, we utilized the full data available to identify the most likely targeted epitopes within the peptides and their HLA presenting molecules (Supplemental Excel Files 1–3). The ELISpot response data provided an estimate of the level of the response to each peptide tested; the ICS response data were used to determine whether a given ELISpot peptide response was mediated by CD4^+^ T cells, CD8^+^ T cells, or both; HLA typing and peptide reactivity were then coupled with known HLA-appropriate epitopes in the HIV database and with Immune Epitope Database (IEDB) predictions to identify likely targeted epitopes within positive peptides; and finally, patterns of sensitivity and resistance across epitope variants were considered. A detailed summary of each T cell response is provided in the Supplemental Excel Files 1–3, and a condensed version of these tables summarizing distinct responses to each epitope variant is shown in Figure 3 and Supplemental Figure 7.

**Figure 5:**
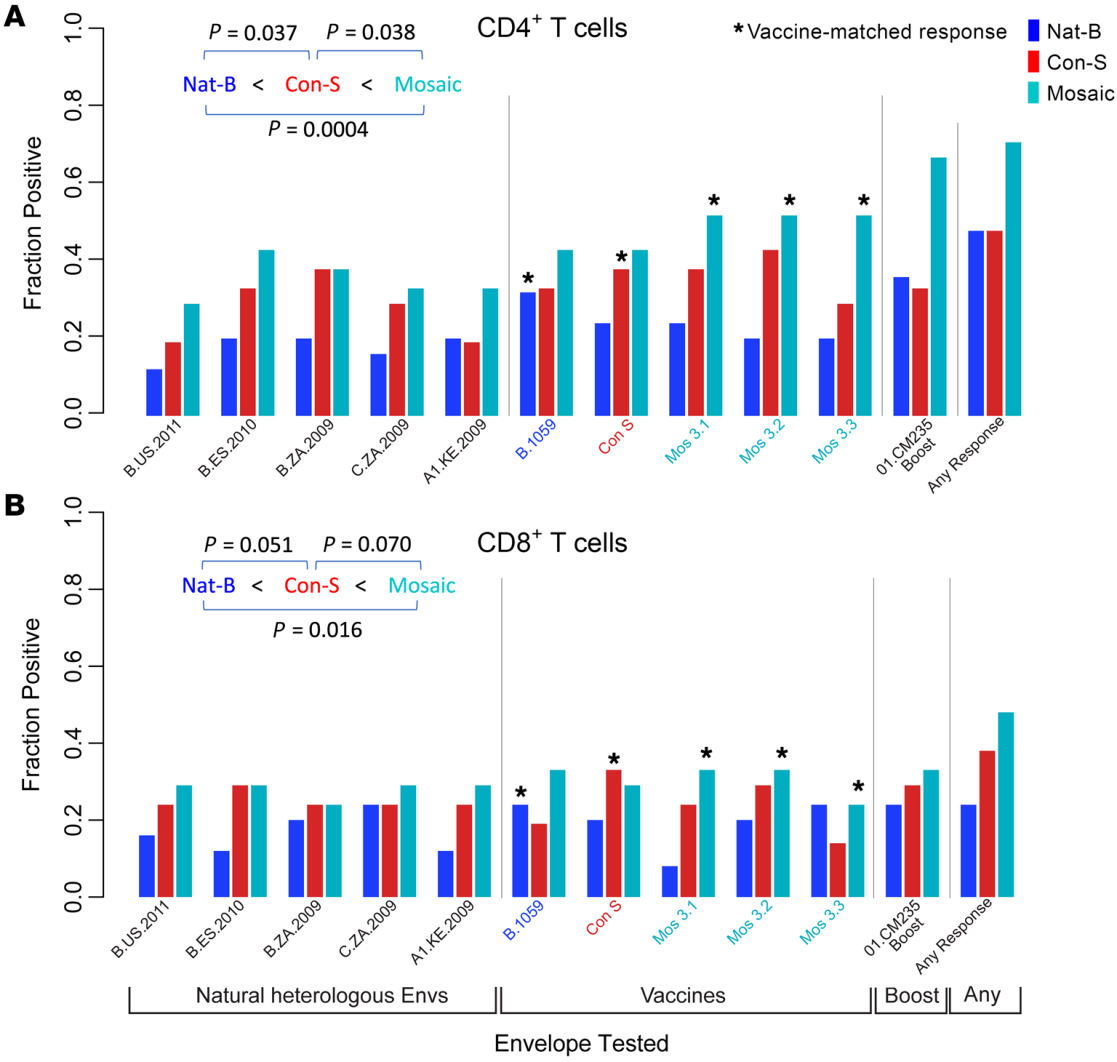
Fraction of individuals that made detectable T cell responses. CD4^+^ (**A**) or CD8^+^ (**B**) T cell responses to 5 heterologous natural variants, and the prime and boost vaccine antigens. Responses were measured using ELISpot as described in Figure 3, using subsequent single-peptide ICS experiments to determine whether it was a CD4^+^ or CD8^+^ T cell response. The numbers of responders to the Envs that were incorporated in the vaccines tended to be among the highest, as expected, and are marked by asterisks. Having 3 Envs in the cocktail tended to enhance the number of responders; for example, a higher fraction of the mosaic than of the Nat-B group made CD4^+^ and CD8^+^ T cell responses to B.1059, the vaccine prime used in the Nat-B group. The mosaic prime group more frequently responded, and more frequently made responses that could recognize the boost antigen, 01.CM235. A paired 2-tailed *t* test was used to compare the fraction of individuals able to make a detectable response to each of the 5 heterologous Envs tested, and the pairwise comparisons for the vaccine groups are provided in the figure. The responses against all heterologous Envs are low.

To examine variant recognition, we computed the fraction of individuals in each group that were able to make a detectable CD4^+^ or CD8^+^ T cell response to each of the vaccines and heterologous variants tested (Figure 5). As noted above for the ICS results, a large fraction of individuals did not make any detectable T cell response, although more mosaic-primed vaccinated individuals had detectable responses than CON-S or Nat-B priming (Figure 2B and Figures 3–5). Again, as seen in the ICS data (Figure 2B and Supplemental Figure 7), CD4^+^ T cell responses were more frequent than CD8^+^ T cell responses. Boost-targeting responses against CM235 were triggered more frequently by the mosaic prime (67% of vaccinees made a CD4^+^ T cell response that recognized CM235, 33% made a CD8^+^ T cell response) than by either the CON-S (33% CD4^+^, 29% CD8^+^) or the Nat-B prime (36% CD4^+^, 24% CD8^+^), but this did not reach statistical significance (Figure 5). Mosaic-primed vaccinees tended to make comparable or better responses to all of the vaccine-matched peptides: mosaic, B.1059, and CON-S.

A key question was how frequently an individual’s vaccine-elicited Env T cell responses were able to cross-react with natural variants. A significantly higher proportion of mosaic vaccine–primed individuals mounted a CD4^+^ T cell (*P* = 0.0004, paired 2-tailed *t* test) or CD8^+^ T cell (*P* = 0.016) response that could recognize the 5 heterologous viruses included in the study than did individuals in the Nat-B–primed group; the CON-S group had intermediate numbers of cross-reactive responses (Figure 5). However, while the mosaic vaccine prime significantly enhanced the number of responses to heterologous variants over the Nat-B and CON-S primes, the fraction of vaccinated individuals able to generate even a single response to a typical heterologous strain was markedly low. For CD4^+^ T cells, averaged over the 5 heterologous Env variants, only 35.2% of mosaic-primed individuals had a detectable response. Only 27.6% of CON-S–primed individuals and 18% of Nat-B had a detectable response (Figure 5). For CD8^+^ T cells, the values were even lower, and only a very small number of vaccinees had detectable responses to the 5 natural variants. Given that having more than 1 cross-reactive response may be critical to confer protective effects (2, 13, 15), we also looked at the capacity of the different vaccine groups to have 2 or more responses to natural variants; on average, 15.4% of the mosaic group, 11.8% of the CON-S, and 4% of the Nat-B vaccine group made 2 responses that were cross-reactive with a given natural variant among the 5 tested (Supplemental Figure 8).

### Epitope “hot spots” across treatment groups were likely driven by participant HLA genotype.

**Figure 6:**
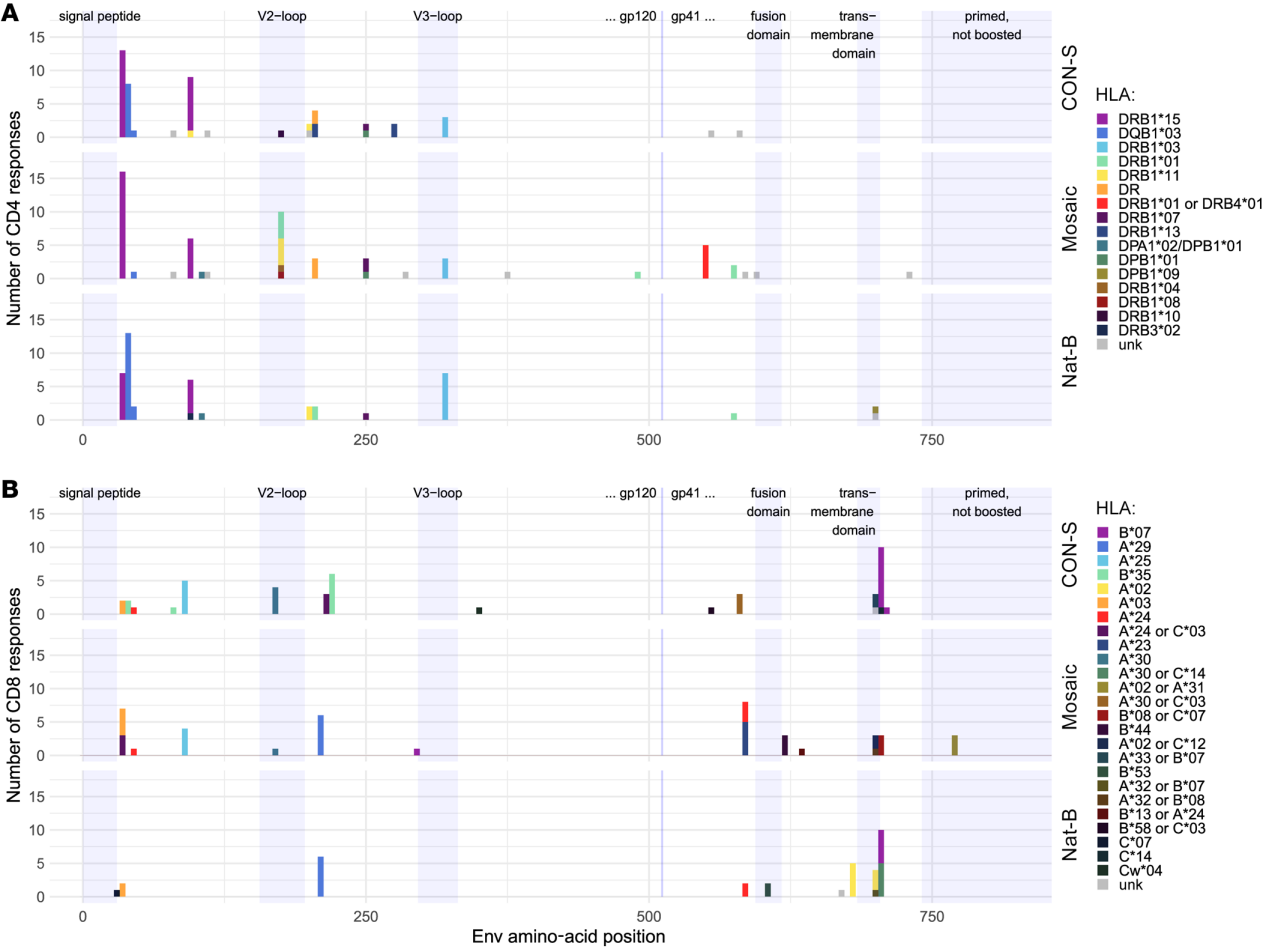
HLA-dependent shared epitope targeting. For each of the 3 vaccine groups, the numbers of predicted T cell epitopes are shown (*y* axis) at each location along the Env protein (HXB2 numbering). The likely presenting HLA is indicated by colors. (**A**) CD4^+^ T cell responses. (**B**) CD8^+^ T cell responses. CD4^+^ T cell responses tend to cluster in the N-terminal region, and these are most often presented by HLA-DRB1*15 and -DQB1*03.

The CD4^+^ T cell responses for all 3 vaccine groups tended to be highly clustered near the N-terminal of the Env protein, with many responders targeting a small number of specific epitopes in the C1 region of the protein. Between HXB2 positions 30–48 (a region spanning 2 overlapping peptides, with an IEDB predicted DRB1*1501 core epitope region in each one, ENLWVTVYY or VTVYYGVPV) and 90–104 (IEDB predicted core FNMWKNNMV) there are 2 immunodominant epitope response regions both associated with known HLA-DRB1*15–restricted epitopes (Figure 6). Twenty-eight percent of study participants carry DRB1*15 or *1503 alleles, and responses to these peptides were highly enriched among these individuals (*P* = 0.0002 and *P* = 0.0008 for the regions 30–48 and 90–104, respectively, Fisher’s exact test); 56% of them target one or both of these epitopes (see Supplemental Excel File 1 for details). These DRB1*15 epitopes were also heavily targeted in RV144 (44). Between these 2 epitopes is an HLA-DQB1*03 epitope that is also very frequently targeted, 38–44 (IEDB predicted core VYYGVPV). Yet another frequently targeted peptide is in the V3 loop, 313–321 (core IIGDIRQAH), presented by DRB1*0301. Much of the total response observed in this study is focused on this small set of epitopes; people carrying these appropriate HLA types are enriched for being CD4^+^ T cell responders in the Env-based vaccine scenario evaluated here.

Another immunodominant region is located in the highly variable V2 loop, Env peptide 173–187, YALFYRLDVVPIDDN (Figure 6). This peptide includes a promiscuous HLA-DR binding peptide (45) and was also commonly recognized in RV144 samples (43% of those tested; ref. 44). This is a highly variable region, and yet mosaic priming serves to stimulate a consistent boost response (Supplemental Figure 9). All 3 of the very distinctive mosaic proteins can trigger a response to this region, and 10 individuals in the mosaic group responded to this epitope; in contrast, only a single CON-S–primed participant responded, and no responses to this region were observed in the Nat-B group. It is an interesting example of a case in which 3 very distinctive variant forms are available in the prime, and each of them can contribute to the overall vaccine response of the group (Supplemental Figure 9). It has been hypothesized that responses to this region may be advantageous because of the close proximity to a critical B cell epitope region targeted by antibodies in RV144 (44, 46) thought to have contributed to RV144’s protective effect (10, 47), such as the antibodies CH58 and CH59 (Supplemental Figure 9) (48).

### CD4^+^ T cell response breadth and magnitude correlated with increased magnitude of binding antibodies.

**Figure 7:**
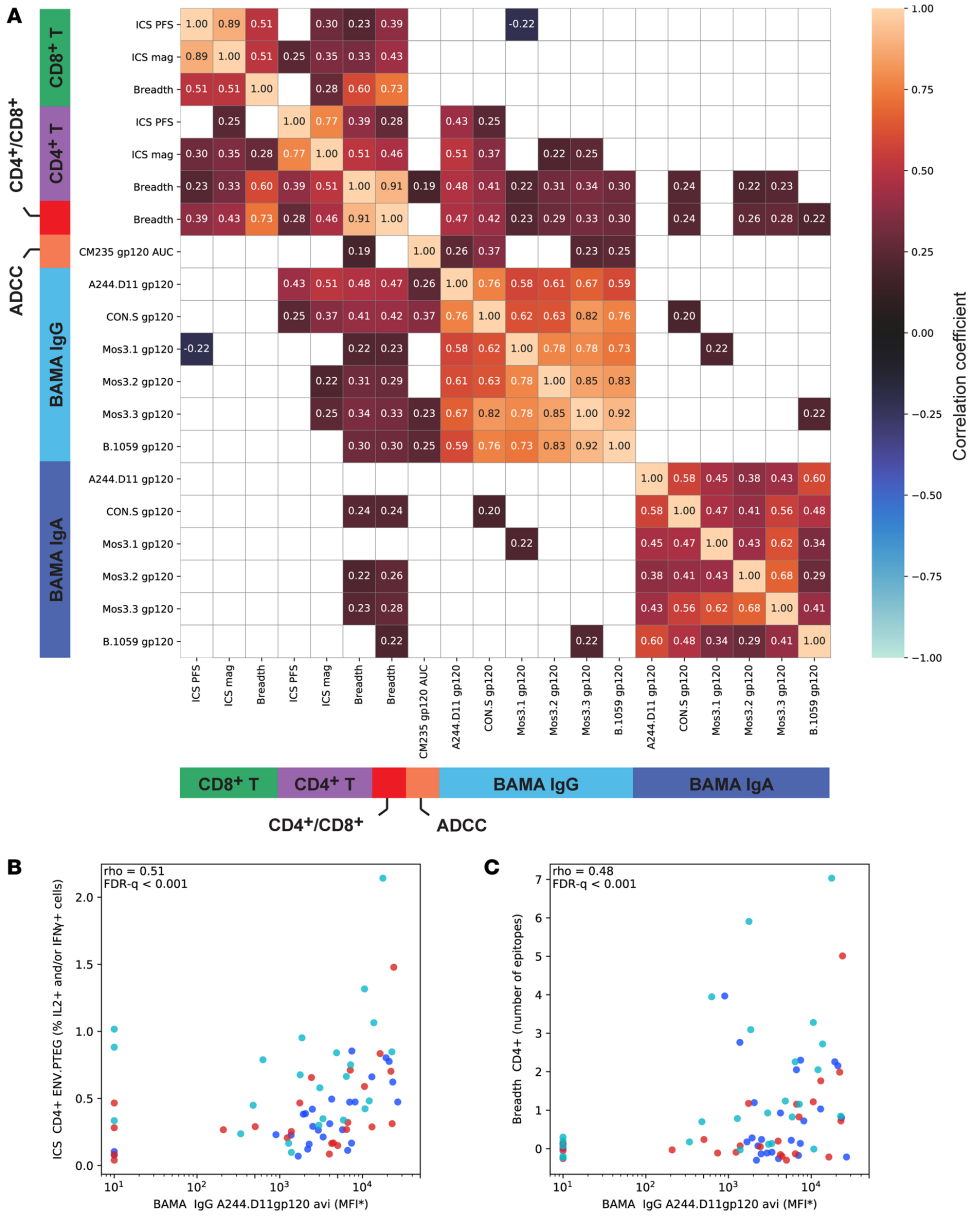
Immune response correlations at 2 weeks after second MVA. Rank-based correlation coefficients were estimated for pairs of immune response measures from participants in all 3 vaccine groups (Nat-B, CON-S, and mosaic), adjusted for treatment group (63). (**A**) Correlation coefficients annotate each pair of measures, with color also indicating the strength of the correlation; correlations with FDR-adjusted *q* value greater than 0.2 are plotted as white. Immune responses are grouped by type: T cell epitope breadth, T cell ICS, IgA binding antibody, ADCC, and IgG binding antibody. Two strong correlations are plotted: (**B**) between CD4^+^ T cell response and BAMA IgG response to A244 gp120 and (**C**) between CD4^+^ T cell breadth and BAMA IgG response to A244 gp120. Correlations include participants from the Nat-B (blue), CON-S (red), and mosaic (teal) groups and were adjusted for multiple comparisons across all pairwise immune response tests. MFI*, background-subtracted median fluorescence intensity.

To better understand the implications of the increased CD4^+^ T cell breadth observed in the mosaic group, we evaluated how CD4^+^ T cell breadth correlated with other vaccine-induced immune responses. We found that CD4^+^ T cell breadth was moderately correlated with the magnitude of the Env-specific CD4^+^ T cell responses (ICS), both measured 2 weeks after the fifth vaccination (rank correlation, ρ = 0.51, FDR-*q* < 0.001). We also found that the Env-specific CD4^+^ T cell response magnitude and breadth correlated with the magnitude of the contemporaneous antibody responses, in particular with the binding antibody response to A244 gp120 (*r* = 0.51 and *r* = 0.48, respectively, both FDR-*q* < 0.001; Figure 7).

## Discussion

Sequence diversity of HIV-1, particularly in Env, continues to present a daunting problem for both antibody and T cell response cross-reactivity. The combination of 3 complementary Env vaccine antigens can provide approximately twice as much coverage of potential T cell epitopes (PTEs) in the circulating global population as the use of a single Env, such that roughly 40% of the PTEs in the population are exactly matched by the vaccine rather than only about 20% (Supplemental Figure 10). In this study, we evaluated how well this theoretical advantage translated into T cell and antibody responses.

We found that the mosaic immunogen more consistently primed broader Env-specific CD4^+^ T cell responses. We also found that broader CD4^+^ T cell responses correlated with vaccine-matched antibody titers. However, despite the overall correlation between CD4^+^ T cell responses and antibody titers, vaccination with the mosaic immunogen did not lead to higher antibody titers. The ability of Env vaccine–induced T cell responses to interact with heterologous isolates was significantly improved in the group using the mosaic prime versus using CON-S or Nat-B Env prime, but responses to heterologous Envs were still very limited in all groups. Underlying the improved breadth were several dominant HLA-restricted T cell responses and a variety of subdominant responses. Still, only a small number of vaccinees elicited T cell responses that could interact with the 5 heterologous Envs tested. For CD4^+^ T cells, on average only 35% of mosaic-vaccinated participants could recognize a given heterologous variant, 28% of CON-S, and only 18% of Nat-B; for CD8^+^ T cells it was 28%, 25%, and 15%, respectively.

These novel vaccine candidates were safe, well tolerated, and immunogenic in this first-in-human evaluation of the trivalent mosaic, CON-S, and Nat-B DNA immunogens. Env-specific antibody responses were induced after 3 DNA immunizations in nearly all participants, were boosted by the MVA-CMDR vaccine, and persisted for 6 months following the last vaccination. Importantly, HIV-specific CD4^+^ T cell responses were also consistently elicited after the DNA immunizations and at a higher magnitude and response rate among the mosaic group compared with the Nat-B group in particular. After the heterologous MVA boosts, this difference was no longer observed. However, when we mapped the Env-specific T cell responses after the MVA boosts, we detected a significantly higher breadth of T cell epitopes targeted among the mosaic group. We found that this difference in breadth was specifically driven by an increased breadth of CD4^+^ T cell epitopes and that it correlated with the overall CD4^+^ T cell response and polyfunctionality. Responses to CD8^+^ T cells were much lower, but this was not unexpected given past experience with this vaccine delivery strategy using a DNA prime, MVA boost (49–51). This delivery strategy was specifically chosen to favor CD4^+^ T cell responses to enable exploration of their response breadth and cross-reactivity because of their critical role in support of antibody responses. Importantly, Env-specific CD4^+^ T cell breadth also correlated with the magnitude of Env-specific binding antibodies (Figure 7).

These findings suggest that CD4^+^ T cell epitope breadth may be important for induction of functional Env-specific humoral responses in a heterologous prime-boost regimen. One hypothesis is that broader T cell epitopes are primed with the DNA mosaic vaccine and that this corresponds to increased epitope recognition expanded by the heterologous boost immunogen. While we were unable to determine the T cell breadth after the DNA-prime vaccinations, overall Env-specific CD4^+^ T cell response magnitudes, rates, and polyfunctionality were highest in the mosaic group, consistent with higher T cell breadth. These data are reminiscent of the findings in nonhuman primate models in which mosaic immunogens elicited increased humoral responses and T cell response breadth (37, 52) and provided partial protection against SHIV challenges (27).

Using complete peptide sets spanning 5 diverse heterologous variants representative of Envs circulating in the global population, we found that a significantly higher fraction of individuals in the mosaic group had at least one CD4^+^ T cell response that could interact with a heterologous strain — approximately 30%–40%, versus 20% or less for the Nat-B group (Figure 5 and Supplemental Figure 10). The mosaic vaccine yielded more cross-reactive responses, a proof of principle that the optimized trivalent combination yielded improved breadth and cross-reactive potential against circulating variants compared with a single natural variant. Still, most participants did not mount a detectable cross-reactive response to the 5 heterologous Envs even in the mosaic group. Improved methods for induction of cross-reactive T cell responses may be essential for vaccine success moving forward. The inclusion of conserved regions of the HIV-1 proteome in vaccine designs, as well as polyvalent vaccine designs that capture the most common circulating forms of epitopes to enhance the cross-reactive potential of T cell responses, may both be needed for vaccine success (2, 34).

Our DNA vaccines expressed HIV-1 Env in order to test the mosaic antigen concept, and Env-expressing mosaic vaccines are also being evaluated in the ongoing Mosaico (HVTN 706; ClinicalTrials.gov NCT03964415) efficacy trial and the recently completed Imbokodo (HVTN 705; NCT03060629) study (8). The HVTN 706/MOSAICO efficacy trial recently reported that the vaccine regimen was not effective in preventing HIV infections (53). In the primary analysis, Imbokodo was found to have an estimated vaccine efficacy of 14% over 7–24 months, which suggests there may have been only a limited effect with the mosaic insert vaccine tested (8). The Imbokodo study design and our study design (HVTN 106) had in common the use of a single natural strain as a heterologous boost following a mosaic prime, namely a clade C protein boost in Imbokodo and a CRF01 variant delivered by MVA here. It is possible that these boosts favor type-specific responses, and we observed that T cell responses against the boost antigen were significantly higher than those against the heterologous antigens tested.

Overall, the mosaic HIV-1 vaccines consistently induced superior Env-specific cellular immune responses especially as compared with the natural subtype B (Nat-B) vaccine, and these responses were significantly boosted following inoculation with a cross-clade heterologous MVA-CMDR. However, our findings highlight the limited ability of these Env antigens to elicit responses able to interact with heterologous variants. While the mosaic vaccines offered significant improvement over the consensus or natural subtype vaccines, the capacity for vaccine-elicited T cells to recognize representative heterologous circulating strains needs to be improved. This study supports the concept of mosaic Env DNA vaccines as a possible “universal” prime in combination with improved heterologous Env boost immunogens for induction of robust Env-specific cross-reactive CD4^+^ T cell help and functional antibodies. The novel trivalent mosaic immunogen and the mosaic immunogen concept warrant additional investigation as components of vaccines for HIV-1. In view of the low levels of cross-reactive responses, however, further evaluation is needed to determine the optimal prime-boost regimens for these novel immunogens.

## Methods

### Vaccines and safety assessments

Details on the construction of the vaccines used and the safety assessments following vaccination are given in Supplemental Methods.

### Participants and study design

This study was a multicenter, randomized, double-blind, placebo-controlled trial to evaluate safety and immunogenicity of 3 different regimens of a DNA prime (at weeks 0, 4, and 8) followed by boosting with MVA-CMDR at 10^8^ PFU/mL (at weeks 16 and 32). The study schema is presented in Figure 1. Further details are available in Supplemental Methods.

### Immunogenicity studies

Immunogenicity assessments were performed on samples collected at weeks 0, 10, 18, 34, and 56. All immunogenicity assays were performed in a blinded fashion in Good Clinical Laboratory Practice–compliant laboratories. Binding antigen multiplex assays (BAMA) were performed with sera to assess HIV-specific binding antibodies against 3 diverse natural heterologous Envs, 92UG937 (clade A), UK7LN (clade B), and C97ZA.012 (clade C), and against 3 vaccine-matched mosaic gp140 trimers (9, 54, 55).

#### Antibody-dependent cellular cytotoxicity GranToxiLux assay.

Participant sera were incubated with effector cells and gp120-coated target cells as described previously (56). Antibody-dependent cellular cytotoxicity (ADCC) was quantified as net percentage granzyme B activity, which is the percentage of target cells positive for GranToxiLux (GTL), an indicator of granzyme B uptake, minus the percentage of target cells positive for GTL when incubated with effector cells in the absence of serum. Further details are available in Supplemental Methods.

#### ADCC luciferase assay.

AADCC-mediated antibody responses were also measured by an ADCC Luciferase assay from CM235-2.LucR.T2A/293T/17 infectious molecular clone–infected (IMC-infected) target cells. Participant sera in addition to control sera were incubated with IMC-infected cells and tested in a 96-well plate. ADCC was detected through the use of ViviRen luminescence (Promega). Further details are available in Supplemental Methods.

#### Neutralization assay.

Neutralizing antibody assays in TZM-bl cells were performed as described previously (57, 58). The data were calculated as a reduction in luminescence compared with the fluorescence in the control wells and are reported as serum dilution, which equals the 50% inhibitory dose (ID_50_).

#### Intracellular cytokine staining assay.

Flow cytometry was used to examine HIV-1–specific CD4^+^ and CD8^+^ T cell response rates and magnitudes using a previously published validated intracellular cytokine staining (ICS) assay (59). PBMCs obtained at visit 7 (week 10), corresponding to 2 weeks after the third vaccination, visit 12 (week 34), corresponding to 2 weeks after the fifth (last) vaccination, and visit 15 (week 56), corresponding to 6 months after the fifth (last) vaccination, were evaluated. The peptide pools used for this were global potential T cell epitope (PTEg) pools Env-1-PTEg, Env-2-PTEg, and Env-3-PTEg (60). Further details are available in Supplemental Methods.

#### T cell epitope mapping.

IFN-γ ELISpot assays were performed to map the epitopes targeted by the HIV-specific T cells and to assess the relative magnitude of the responses using 15-mer peptides overlapping by 11 aa matching the Mos-1, Mos-2, Mos-3, CON-S, B.1059, and CRF01.CM235 Env sequences, as well as peptides matched to 5 heterologous circulating HIV-1 Env sequences that were selected to represent diverse transmitted founder viruses from different clades and nations. Three subtype B viruses were included to enable exploration of intra- versus inter-subtype responses to the natural B clade prime vaccine B.1059. The Env sequences used were B.US.2011 (GenBank accession no. KC473833), B.ZA.2009 (HQ595755), B.ES.2010 (KC473843), A1.KE.2009 (HQ540689), and C.ZA.2009 (HQ595760); taken together, these 5 variants were representative of the PTE diversity found in the HIV-1 database relative to the vaccine antigens (Supplemental Figure 1). Further details are available in Supplemental Methods.

#### HLA class I and II genotyping.

HLA typing was carried out on approximately 1 × 10^6^ residual PBMC cell pellets using the ScisGo HLA v6 typing kit (Scisco Genetics Inc.). Briefly, the method uses a DNA isolation and amplicon-based 2-stage PCR, followed by sample pooling and sequencing using a MiSeq v2 PE500 (Illumina) (61, 62).

### Statistics

Criteria for positive neutralizing antibody responses were titer >10 and for Env ELISA responses were titer ≥100; antibody responses below the lower limit of quantitation (LLOQ) of the assay were assigned the numeric value of the LLOQ. Positivity assessments for the ICS assay and the IFN-γ ELISpot as well as details of epitope determination, analysis of epitope breadth, and statistical testing are described in Supplemental Methods. Tests with a 2-sided *P* value less than 0.05 were considered significant.

### Study approval

The protocol was approved by the institutional review boards and biosafety committees at all sites (Brigham and Women’s Hospital; Fred Hutchinson Cancer Center; San Francisco Department of Public Health; The Fenway Institute; University of Alabama at Birmingham; Emory University; and Centre Hospitalier Universitaire Vaudois), and written informed consent was obtained from each participant. The study was registered at ClinicalTrials.gov (NCT02296541).

## Author contributions

KWC, AFG, SRW, WF, BFH, BTK, and LRB wrote the initial draft of the manuscript. KY, NF, DEM, SCD, DEG, WF, EEG, HM, GF, GDT, DCM, PBG, and MJM performed the experiments and analyses. SRW, MLE, JM, HS, KHM, PAG, SE, GP, JH, and LRB conducted the clinical trial. MLR, NLM, MNP, BFH, and BTK designed the vaccines tested. All authors reviewed and approved the final manuscript.

## Supplementary Material

Supplemental data

Trial reporting checklists

ICMJE disclosure forms

Supplemental data set 1

Supplemental data set 2

Supplemental data set 3

Supplemental data file: HVTN106-VaccinesPeptideStrains (fasta file)

## Version 1 02/15/2023

Electronic publication

## References

[1] Taylor BS (2008). The challenge of HIV-1 subtype diversity. N Engl J Med.

[2] Korber B (2017). Polyvalent vaccine approaches to combat HIV-1 diversity. Immunol Rev.

[3] Buchbinder SP (2008). Efficacy assessment of a cell-mediated immunity HIV-1 vaccine (the Step Study): a double-blind, randomised, placebo-controlled, test-of-concept trial. Lancet.

[4] Flynn NM (2005). Placebo-controlled phase 3 trial of a recombinant glycoprotein 120 vaccine to prevent HIV-1 infection. J Infect Dis.

[5] Gray GE (2021). Vaccine efficacy of ALVAC-HIV and bivalent subtype C gp120-MF59 in adults. N Engl J Med.

[6] Hammer SM (2013). Efficacy trial of a DNA/rAd5 HIV-1 preventive vaccine. N Engl J Med.

[7] Rerks-Ngarm S (2009). Vaccination with ALVAC and AIDSVAX to prevent HIV-1 infection in Thailand. N Engl J Med.

[8] https://www.jnj.com/johnson-johnson-and-global-partners-announce-results-from-phase-2b-imbokodo-hiv-vaccine-clinical-trial-in-young-women-in-sub-saharan-africa.

[9] Haynes BF (2012). Immune-correlates analysis of an HIV-1 vaccine efficacy trial. N Engl J Med.

[10] Gottardo R (2013). Plasma IgG to linear epitopes in the V2 and V3 regions of HIV-1 gp120 correlate with a reduced risk of infection in the RV144 vaccine efficacy trial. PLoS One.

[11] Tolbert WD (2021). Recognition patterns of the C1/C2 epitopes involved in Fc-mediated response in HIV-1 natural infection and the RV144 vaccine trial. mBio.

[12] Lin L (2015). COMPASS identifies T-cell subsets correlated with clinical outcomes. Nat Biotechnol.

[13] Janes HE (2017). Higher T-cell responses induced by DNA/rAd5 HIV-1 preventive vaccine are associated with lower HIV-1 infection risk in an efficacy trial. J Infect Dis.

[14] Janes H (2012). MRKAd5 HIV-1 Gag/Pol/Nef vaccine-induced T-cell responses inadequately predict distance of breakthrough HIV-1 sequences to the vaccine or viral load. PLoS One.

[15] Janes H (2013). Vaccine-induced gag-specific T cells are associated with reduced viremia after HIV-1 infection. J Infect Dis.

[16] Walker BD, Yu XG (2013). Unravelling the mechanisms of durable control of HIV-1. Nat Rev Immunol.

[17] Kunwar P (2013). Superior control of HIV-1 replication by CD8+ T cells targeting conserved epitopes: implications for HIV vaccine design. PLoS One.

[18] Kiepiela P (2007). CD8+ T-cell responses to different HIV proteins have discordant associations with viral load. Nat Med.

[19] Mothe B (2011). Definition of the viral targets of protective HIV-1-specific T cell responses. J Transl Med.

[20] Murakoshi H (2018). CD8^+^ T cells specific for conserved, cross-reactive Gag epitopes with strong ability to suppress HIV-1 replication. Retrovirology.

[21] Ondondo B (2016). Novel conserved-region T-cell mosaic vaccine with high global HIV-1 coverage is recognized by protective responses in untreated infection. Mol Ther.

[22] Zou C (2019). Effective suppression of HIV-1 replication by cytotoxic T lymphocytes specific for Pol epitopes in conserved mosaic vaccine immunogens. J Virol.

[23] Rolland M (2011). Genetic impact of vaccination on breakthrough HIV-1 sequences from the STEP trial. Nat Med.

[24] Hertz T (2013). HIV-1 vaccine-induced T-cell responses cluster in epitope hotspots that differ from those induced in natural infection with HIV-1. PLoS Pathog.

[25] Martins MA (2017). Vaccine-induced immune responses against both Gag and Env improve control of simian immunodeficiency virus replication in rectally challenged rhesus macaques. PLoS Pathog.

[26] Roederer M (2014). Immunological and virological mechanisms of vaccine-mediated protection against SIV and HIV. Nature.

[27] Barouch DH (2013). Protective efficacy of a global HIV-1 mosaic vaccine against heterologous SHIV challenges in rhesus monkeys. Cell.

[28] Liu J (2009). Immune control of an SIV challenge by a T-cell-based vaccine in rhesus monkeys. Nature.

[29] Petitdemange C (2019). Vaccine induction of antibodies and tissue-resident CD8+ T cells enhances protection against mucosal SHIV-infection in young macaques. JCI Insight.

[30] Barouch DH (2018). Evaluation of a mosaic HIV-1 vaccine in a multicentre, randomised, double-blind, placebo-controlled, phase 1/2a clinical trial (APPROACH) and in rhesus monkeys (NHP 13-19). Lancet.

[31] Arunachalam PS (2020). T cell-inducing vaccine durably prevents mucosal SHIV infection even with lower neutralizing antibody titers. Nat Med.

[32] Fischer W (2007). Polyvalent vaccines for optimal coverage of potential T-cell epitopes in global HIV-1 variants. Nat Med.

[33] Gao F (2004). Centralized immunogens as a vaccine strategy to overcome HIV-1 diversity. Expert Rev Vaccines.

[34] Korber B, Fischer W (2020). T cell-based strategies for HIV-1 vaccines. Hum Vaccin Immunother.

[35] Korber BT (2009). 2009. T-cell vaccine strategies for human immunodeficiency virus, the virus with a thousand faces. J Virol.

[36] Gaschen B (2002). Diversity considerations in HIV-1 vaccine selection. Science.

[37] Barouch DH (2010). Mosaic HIV-1 vaccines expand the breadth and depth of cellular immune responses in rhesus monkeys. Nat Med.

[38] Santra S (2010). Mosaic vaccines elicit CD8^+^ T lymphocyte responses that confer enhanced immune coverage of diverse HIV strains in monkeys. Nat Med.

[39] Nickle DC (2003). Consensus and ancestral state HIV vaccines. Science.

[40] Haynes BF, Bradley T (2015). Broadly neutralizing antibodies and the development of vaccines. JAMA.

[41] Elizaga ML (2013). Prospective surveillance for cardiac adverse events in healthy adults receiving modified vaccinia Ankara vaccines: a systematic review. PLoS One.

[42] Fiore-Gartland A (2016). Pooled-peptide epitope mapping strategies are efficient and highly sensitive: an evaluation of methods for identifying human T cell epitope specificities in large-scale HIV vaccine efficacy trials. PLoS One.

[43] Walsh SR (2016). Vaccination with heterologous HIV-1 envelope sequences and heterologous adenovirus vectors increases T-cell responses to conserved regions: HVTN 083. J Infect Dis.

[44] Ratto-Kim S (2015). Identification of immunodominant CD4-restricted epitopes co-located with antibody binding sites in individuals vaccinated with ALVAC-HIV and AIDSVAX B/E. PLoS One.

[45] Fonseca SG (2006). Identification of novel consensus CD4 T-cell epitopes from clade B HIV-1 whole genome that are frequently recognized by HIV-1 infected patients. AIDS.

[46] Steede NK (2013). Shaping T cell – B cell collaboration in the response to human immunodeficiency virus type 1 envelope glycoprotein gp120 by peptide priming. PLoS One.

[47] Karasavvas N (2012). The Thai Phase III HIV type 1 vaccine trial (RV144) regimen induces antibodies that target conserved regions within the V2 loop of gp120. AIDS Res Hum Retroviruses.

[48] Liao HX (2013). Vaccine induction of antibodies against a structurally heterogeneous site of immune pressure within HIV-1 envelope protein variable regions 1 and 2. Immunity.

[49] Bakari M (2011). Broad and potent immune responses to a low dose intradermal HIV-1 DNA boosted with HIV-1 recombinant MVA among healthy adults in Tanzania. Vaccine.

[50] Joachim A (2017). Three-year durability of immune responses induced by HIV-DNA and HIV-modified vaccinia virus Ankara and effect of a late HIV-modified vaccinia virus Ankara boost in Tanzanian volunteers. AIDS Res Hum Retroviruses.

[51] Joachim A (2015). Potent functional antibody responses elicited by HIV-I DNA priming and boosting with heterologous HIV-1 recombinant MVA in healthy Tanzanian adults. PLoS One.

[52] Santra S (2012). Breadth of cellular and humoral immune responses elicited in rhesus monkeys by multi-valent mosaic and consensus immunogens. Virology.

[53] https://www.jnj.com/janssen-and-global-partners-to-discontinue-phase-3-mosaico-hiv-vaccine-clinical-trial.

[54] Tomaras GD (2013). Vaccine-induced plasma IgA specific for the C1 region of the HIV-1 envelope blocks binding and effector function of IgG. Proc Natl Acad Sci U S A.

[55] Tomaras GD (2008). Initial B-cell responses to transmitted human immunodeficiency virus type 1: virion-binding immunoglobulin M (IgM) and IgG antibodies followed by plasma anti-gp41 antibodies with ineffective control of initial viremia. J Virol.

[56] Pollara J (2011). High-throughput quantitative analysis of HIV-1 and SIV-specific ADCC-mediating antibody responses. Cytometry A.

[57] Montefiori DC (2005). Evaluating neutralizing antibodies against HIV, SIV, and SHIV in luciferase reporter gene assays. Curr Protoc Immunol.

[58] Montefiori DC (2009). Measuring HIV neutralization in a luciferase reporter gene assay. Methods Mol Biol.

[59] Moncunill G (2015). OMIP-025: evaluation of human T- and NK-cell responses including memory and follicular helper phenotype by intracellular cytokine staining. Cytometry A.

[60] Li F (2006). Peptide selection for human immunodeficiency virus type 1 CTL-based vaccine evaluation. Vaccine.

[61] Nelson WC (2015). An integrated genotyping approach for HLA and other complex genetic systems. Hum Immunol.

[62] Smith AG (2019). Comparison of sequence-specific oligonucleotide probe vs next generation sequencing for HLA-A, B, C, DRB1, DRB3/B4/B5, DQA1, DQB1, DPA1, and DPB1 typing: toward single-pass high-resolution HLA typing in support of solid organ and hematopoietic cell transplant programs. HLA.

[63] https://www.liebertpub.com/doi/10.1089/aid.2018.5000.abstracts.

[64] Liu Q (2018). Covariate-adjusted Spearman’s rank correlation with probability-scale residuals. Biometrics.

