# Supplementary material for: Trivalent mosaic or consensus HIV immunogens prime humoral and broader cellular immune responses in adults
Source: J Clin Invest. 2023 Feb 15;133(4):e163338. doi: 10.1172/JCI163338 (PMC9927951; doi:10.1172/JCI163338)
Supplement: Supplemental data [file jci-133-163338-s251.pdf]

SUPPLEMENTAL MATERIALS

Trivalent mosaic or consensus HIV immunogens prime humoral and broader cellular immune responses in adults

Kristen W. Cohen[1,*], Andrew Fiore-Gartland[1,*], Stephen R. Walsh[2,3,4,*], Karina Yusim[5,*,†], Nicole Frahm[1,6], Marnie L. Elizaga[1], Janine Maenza[1], Hyman Scott[7], Kenneth H. Mayer[3,8], Paul A. Goepfert[9], Srilatha Edupuganti[10], Giuseppe Pantaleo[11], Julia Hutter[12], Darryl E. Morris[1], Stephen C. De Rosa[1], Daniel E. Geraghty[1], Merlin L. Robb[13], Nelson L. Michael[14], Will Fischer[5], Elena E. Giorgi[5], Harman Malhi[1], Michael N. Pensiero[12], Guido Ferrari[15], Georgia D. Tomaras[15], David C. Montefiori[15], Peter B. Gilbert[1], M. Juliana McElrath[1], Barton F. Haynes[15,¶], Bette T. Korber[5,¶], Lindsey R. Baden[2,3,¶], and the NIAID HVTN 106 Study Group

[1] Vaccine and Infectious Disease Division, Fred Hutchinson Cancer Research Center, Seattle, WA, USA
[2] Division of Infectious Diseases, Brigham and Women's Hospital, Boston, MA
[3] Harvard Medical School, Boston, MA, USA
[4] Center for Virology and Vaccine Research, Beth Israel Deaconess Medical Center, Boston, MA, USA
[5] Theoretical Biology and Biophysics Group, Los Alamos National Laboratory, and the New Mexico Consortium, Los Alamos, NM, USA
[6] Department of Global Health, University of Washington, Seattle, WA, USA
[7] San Francisco Department of Public Health, San Francisco, CA, USA
[8] The Fenway Institute, Fenway Health, Boston, MA, USA
[9] University of Alabama at Birmingham, Birmingham, AL, USA
[10] Emory University, Atlanta, GA, USA
[11] Centre Hospitalier Universitaire Vaudois, Lausanne, Switzerland
[12] Division of AIDS, National Institute of Allergy and Immunology, Bethesda, MD, USA
[13] Henry M. Jackson Foundation for the Advancement of Military Medicine, Bethesda, MD, USA
[14] Walter Reed Army Institute of Research, Silver Spring, MD, USA
[15] Duke Human Vaccine Institute, Duke University, Durham, NC, USA

* These authors contributed equally.
† Deceased.
¶ These authors contributed equally.

SUPPLEMENTAL ACKNOWLEDGMENTS
The NIAID HVTN 106 Study Group: In addition to the authors of this article, members of the NIAID HVTN 106 Study Group include Jill Zeller, Carissa Karg, Gina Escamilla, Gail Broder, Huguette Redinger, David Berger, Kim Louis, Rotrease Reagan, Susan P. Buchbinder, Jane A. Kleinjan, Jon A. Gothing, Brian A. Engelson, Rachel D. Filter, Michael S. Seaman, Raphael Dolin, and Jennifer A. Johnson.

SUPPLEMENTAL METHODS

Vaccines

The three DNA priming vaccines (DNA Nat-B *env*, DNA CON-S *env* and DNA Mosaic *env*) were generated using a DNA backbone derived from pCMV/R (pVRC8400) which was generously donated by the NIAID Vaccine Research Center (Bethesda, MD) and has been used as a backbone for several prior candidate HIV-1 vaccines (1, 2).

The natural clade B gp160 *env* sequence was derived from B.1059 (HV13288), a transmitted/founder (T/F) virus selected because, at the time, it ranked highest in terms of providing the best coverage among natural strains for nonamer (9-mer) potential T cell epitopes (PTEs) in the Los Alamos HIV database (3). B.1059 provided 23% PTE coverage, while typical natural strains cover an average of 15% of PTEs. The CON-S *env* sequence expresses gp160 Env protein from a Group M consensus virus (HV13287) (4). CON-S is a consensus of HIV subtype consensus sequences (31) and is a second-generation M group vaccine design, following CON-6. Both CON-S and CON-6 were able to induce improved T cell responses in mice relative to wildtype proteins (4-6). DNA Mosaic *env* is a trivalent vaccine composed of Mosaic HV13284, HV13285 and HV13286 *env* sequences at a 1:1:1 ratio that express gp160 Env and were optimized as a combination for global coverage (7, 8). A comparison of the PTE coverage of the contemporary 2019 global M group reference alignment from the Los Alamos database for the 3 vaccine antigens is shown in **Figure S10**. Use of the trivalent Mosaic provides perfect matches to 42% of the PTEs in the Env in this global alignment,

The booster vaccine used in this study was a modified vaccinia Ankara (MVA) vectored HIV-1 vaccine (MVA-Chiang Mai Double Recombinant [CMDR]) that has been genetically engineered to express HIV-1 gp150 Env (circulating recombinant form CRF01, an AE recombinant, isolate CM235) and Gag and Pol (integrase-deleted and reverse transcriptase nonfunctional, CRF01 isolate CM240) (9). The gp150 env gene in MVA-CMDR has a truncated cytoplasmic tail, but the transmembrane domain is unaffected (9). MVA-CMDR has previously been tested both alone and in prime-boost regimens with DNA vaccines (10, 11) and was administered at a dose of 1×10^8 pfu. The placebo was 0.9% sodium chloride. The original study design had a matched prime-boost vaccine regimen to enable a more direct test of the mosaic and consensus concepts. Due to manufacturing complications, we opted instead to use a heterologous boost across groups; an advantage of this option was that it provided the opportunity to study the capacity of the immune response to the different primes in terms of the ability to interact with a heterologous virus and be effectively boosted.

All participants were screened to not have been vaccinated within the past 10 years with any smallpox vaccines. While we did not assess the possible impact of pre-existing cross-reactive anti-orthopoxvirus NAbs, pre-existing poxvirus-specific NAbs have been found to have minimal impact on immune responses elicited when MVA is used as the primary immunogen (12) and as a vector (13, 14).

Participants and Study Design

This study was a multicenter, randomized, double-blind, placebo-controlled trial to evaluate safety and immunogenicity of three different regimens of a DNA prime (at weeks 0, 4 and 8) followed by boosting with MVA-CMDR at 10^8 pfu/mL (at weeks 16 and 32). Study participants were healthy HIV-negative volunteers between the ages of 18 and 50 who were at low risk for acquiring HIV as per standard criteria (15). The protocol was approved by the institutional review boards and biosafety committees at all sites and written informed consent was obtained from each participant. The study was registered at ClinicalTrials.gov (NCT02296541). The study schema is presented in **Figure 1**. DNA vaccines were given by Biojector 2000® (Biojector, Inc, Bedminster, NJ) into the deltoid muscle except for 16 participants at the Lausanne site due to manufacturing issues; these participants received their DNA vaccines by needle and syringe in the deltoid muscle. MVA-CMDR was given by needle and syringe in the deltoid muscle. A subset of participants at the Seattle and Boston sites underwent leukapheresis for detailed analysis of preexisting memory CD4+ T cells (16).

Safety Assessments

To assess safety, participants were provided a diary card on which they recorded local and systemic reactogenicity for 7 days post-vaccination. Safety laboratory studies were assessed on days 14, 42, 168, 182, and 365 and included complete blood count, serum chemistries, and urinalysis. All participants potentially capable of pregnancy had a negative pregnancy test prior to each vaccination. Reactogenicity (solicited adverse events [AEs]) and unsolicited AEs were assessed as per the NIAID Division of AIDS Table for Grading the Severity of Adult and Pediatric Adverse Events (DAIDS AE

Grading Table), Version 1.0, December 2004 (Clarification August 2009), available on the RCC website at http://rcc.tech-res-intl.com.

Immunogenicity Studies

Antibody Dependent Cellular Cytotoxicity (ADCC) GranToxiLux (GTL) assay

Participant sera were incubated with effector cells and gp120-coated target cells as described previously (17). ADCC was quantified as net percent granzyme B activity which is the percent of target cells positive for GTL, an indicator of granzyme B uptake, minus the percent of target cells positive for GTL when incubated with effector cells in absence of serum. Flow cytometry was used to quantify the frequency of granzyme B-positive cells. For each subject at each time point, percent granzyme B activity was measured at six dilution levels: 50, 250, 1250, 6250, 31,250 and 156,250 for each antigen. Peak net percent granzyme B activity was defined as the maximum activity across the levels ("peak activity"). Peak activity less than 0% was set to 0%. A positive response was defined as peak activity greater than or equal to 8%.

ADCC luciferase assay

ADCC-mediated antibody responses as measured by ADCC Luciferase from CM235-2.LucR.T2A/293T/17 Infectious Molecular Clone (IMC)-infected target cells. Participant sera in addition to control sera were incubated with IMC-infected cells and tested in a 96- well plate. ADCC was detected through the use of Viviren luminescence. One positive control in duplicate and one standardized negative control in duplicate were used per plate. The readout is reduction in Relative Luminescence Units (RLU), referred

to as Percentage Specific Killing (18, 19). For each sample, percent specific killing was measured in duplicate wells and percent loss Luciferase activity was calculated relative to control wells for each experimental well and averaged over wells within subject, time point, and dilution. The baseline-subtracted average percent loss activity was considered a positive response when peak baseline-subtracted activity was greater than or equal to 10% for either the 1:50 or 1:250 dilution.

Intracellular cytokine staining (ICS) assay

Flow cytometry was used to examine HIV-1-specific CD4+ and CD8+ T cell response rates and magnitudes using a previously published validated intracellular cytokine staining (ICS) assay (20). PBMC obtained at visit 7 (wk 10), corresponding to 2 weeks after the 3rd vaccination, visit 12 (wk 34), corresponding to 2 weeks after the 5th (last) vaccination, and visit 15 (wk 56), corresponding to 6 months after the 5th (last) vaccination were evaluated. The peptide pools used for this were global potential T-cell epitope (PTEg) pools Env-1-PTEg, Env-2-PTEg, Env-3-PTEg (21). Previously cryopreserved PBMC were stimulated with synthetic peptide pools and co-stimulation with purified antibodies specific for CD28 and CD49d. As a negative control, cells were stimulated with peptide diluent and co-stimulatory antibodies. As a positive control, cells were stimulated with a polyclonal stimulant, staphylococcal enterotoxin B (SEB). The negative control was plated in two replicates and other stimulations were in singlets. The primary immunogenicity T cell endpoints were CD4+ and CD8+ T cell responses, measured by ICS for IFN-γ and/or IL-2 to any Env peptide pool. Several criteria were used to determine if data from an assay were acceptable and could be statistically

analyzed. First, the blood draw date must have been within the allowable visit window as determined by the protocol. Second, post-infection samples from HIV-infected participants were excluded. Third, PBMC cell viability was required to be 66% or greater on the second day after sample thawing. If not, the sample for that specimen at that time point was retested. If upon retesting the viability remained below this threshold, the ICS assay was not performed, and no data were reported for that time point. Finally, if the average cytokine response for the negative control wells was above 0.1% for either the CD4+ or CD8+ T cells, or the total number of CD4+ and CD8+ T cells was less than 5,000 events, then the sample was retested.

T cell epitope mapping

IFN-γ ELISpot assays were performed to map the epitopes targeted by the HIV-specific T cells and to assess the relative magnitude of the responses using 15-mer peptides overlapping by 11 aa matching the Mos-1, Mos-2, Mos-3, CON-S, B.1059, and CRF01.CM235 Env sequences, as well as peptides matched to five heterologous circulating HIV-1 Env sequences that were selected to represent diverse transmitted founder viruses from different clades and nations. Three subtype B viruses were included to enable exploration of intra- versus inter-subtype responses to the Natural B clade prime vaccine B.1059. The Env sequences used were B.US.2011 (GenBank accession number: KC473833), B.ZA.2009 (HQ595755), B.ES.2010 (KC473843), A1.KE.2009 (HQ540689), and C.ZA.2009 (HQ595760); taken together, these five variants were representative of the PTE diversity found in the HIV-1 database relative to the vaccine antigens (**Figure S1**). All peptides were received as individual peptides,

reconstituted, pooled and validated in both the ELISpot and ICS assays and were used at a final concentration of 1 µg/mL per peptide (Bio-Synthesis, Lewisville, TX). An alignment of the vaccine sequences and the HIV Env sequences used for assessing the cross-reactive potential of T-cell responses is included as supplementary material (see: HVTN106-VaccinesPeptideStrains.fasta).

For Stage 1 testing master pools, IFN-γ ELISpot epitope mapping data were available from 87 participants; 17 participants were excluded for not receiving all vaccines, and one additional participant had a missed visit. Of the 87 remaining participants, 4 participants were completely excluded (1 for viability and 3 for high negative control values). Of the 83 participants with available data for Stage 1 of epitope mapping, 72 participants had a positive response. Of the 72 participants tested in Stage 2 (assessment of mini-pools), one was filtered for being unreliable. 51 participants had a positive response in Stage 2. In Stage 3 (testing of individual 15-mers), two participants were filtered for high background. In all, 80 participants generated reliable data for epitope mapping. There were no positive responses among the 13 participants in the placebo group. Of the 67 participants in the three vaccine groups, 42 (63%) responded to at least one peptide in the final round of mapping with individual 15-mers.

Statistical Methods

ICS assay

To assess positivity for a peptide pool within a T cell subset, a two-by-two contingency

table was constructed comparing the HIV-1-peptide-stimulated and negative control data. The four entries in each table were the number of cells positive for IL-2 and/or IFN-γ and the number of cells negative for IL-2 and/or IFN-γ, for both the stimulated and the negative control data. A one-sided Fisher's exact test was applied to the table, testing whether the number of cytokine-producing cells for the stimulated data was equal to that for the negative control data. Since multiple individual tests (for each peptide pool) are conducted simultaneously, a multiplicity adjustment was made to the individual peptide pool p-values using the discrete Bonferroni adjustment method. If the adjusted p-value for a peptide pool was ≤0.00001, the response to the peptide pool for the T cell subset was considered positive. Because the sample sizes (i.e., total cell counts for the T cell subset) are large, e.g., 100,000 cells, the Fisher's exact test has high power to reject the null hypothesis for very small differences. Therefore, the adjusted p-value significance threshold was chosen stringently (≤0.00001).

IFN-γ ELISpot

In the first round, to determine a positive response to a specific peptide pool, both bootstrap and MIMOSA tests were used. The second and third rounds utilized only bootstrap because of the smaller sample sizes for many minipools (restricted to positives from the first round) and individual peptides. The bootstrap test was used to test the null hypothesis that the mean of the $\log_{10}$ experimental wells was less than or equal to twice the mean of the $\log_{10}$ negative control wells versus the alternative hypothesis that the $\log_{10}$ experimental mean was greater than twice that of the $\log_{10}$ negative control mean. This method adjusts for the multiple peptide pools considered by

calculating step-down maxT adjusted p-values. Peptide pools with adjusted one-sided p-values < 0.05 were declared positive. Positive responses were then examined to confirm biological significance. Namely, in addition to a positive bootstrap call, the mean background-subtracted response for the peptide pool was ≥50 SFC/10^6 PBMC for the peptide pool to be considered positive. The purpose of this criterion was to require a minimal demonstration of biological activity. Two-sided 95% confidence intervals of response rates were calculated using the Wilson score method (22).

Epitope determination

The third round of epitope mapping yielded a list of 15-mers that elicited a positive response for each participant. Since many peptides that elicited responses overlapped, in some cases responses to two overlapping but distinct peptides by a single participant may reflect only one underlying response to an epitope contained within the region common to the overlapping peptides. To provide a consistent and conservative way to identify the minimal number of underlying epitopes that could explain each participant's set of 15-mer responses we applied an overlap criterion that has been previously applied to HVTN epitope mapping data (23): if two or more targeted 15-mers share a region of ≥8 positions, then responses are assumed to be explained by a single epitope in the region of overlap. Following the application of this deterministic algorithm, the minimal response breadth for each participant was quantified; this was then used for a primary comparison of groups.

An alternative analysis of T cell response cross-reactivity was conducted using the full spectrum of rich data available in this study, enabling confident assignment of many

responses to particular HLAs for which optimal epitopes either have been previously determined or are predicted. Using this strategy the most likely epitope within each targeted peptide for each individual response was resolved, and the cross-reactive potential of each response across the heterologous Env proteins was experimentally determined (detailed in the supplementary material). The data incorporated included: i. Participants' HLA typing to determine if either experimentally validated T cell epitopes listed in the HIV immunology database (https://www.hiv.lanl.gov), or HLA-appropriate epitopes based on Immune Epitope Database (IEDB) epitope predictions (http://tools.iedb.org/main/tcell/) were found within the targeted peptides; ii. ICS resolution of CD4+ versus CD8+ T-cell responses; and iii. ELISpot peptide reactivity and response magnitude across variant peptides. Patterns of resistance and sensitivity based on the full sets of variant peptides included in the study were then determined. A comprehensive summary of these integrated data and strategy for epitope resolution is provided as supplementary Information (three supplement information excel files called SupTable_A, B and C; peptides are sequentially ordered by Env positions). These analyses indicated that two independent responses often afforded a more plausible explanation of the data than the assumption of a single response when overlapping peptides were both positive, although in some instances the most likely epitope was in the region of overlap. Statistical comparisons of the number of individuals within vaccine groups that had CD4+ or CD8+ T cell responses that could recognize heterologous variants were made using a two-sided paired t-test.

Analysis of epitope breadth

We computed minimal epitope breadth using four different subsets of the peptide data to address distinct objectives: (1) overall breadth, (2) prime-matched breadth, (3) heterologous breadth relative to 5 distinct heterologous Env variants, and (4) boost-matched breadth. To compute overall breadth the overlap criterion was applied to all the peptide responses of each participant. Prime-matched breadth was computed using peptides matching the DNA vaccines (B.1059, CON-S, Mosaic 1-3). Note that prime-matched breadth is computed using a different peptide set for each group, and the Mosaic set is larger because more variants were included in the priming vaccine. Heterologous breadth was computed using peptides derived from five circulating strains that were not contained in any of the vaccines. In these names the first letter refers to the clade of the virus, the second two letters the two-letter country code, and followed by the year of isolation. Boost-matched breadth was computed using the peptides derived from the CM235 sequence in the MVA vector used as a boost.

Group comparisons

We used non-parametric statistical methods based on the empirical cumulative distribution function to estimate the mean parameter of interest: breadth, the minimal number of responses, and depth, the ability of the response to recognize different HIV-1 variants, for each group. The non-parametric bootstrap was used to compute 95% confidence intervals on each targeted parameter (e.g., within group, mean breadth). To do this we formed bootstrap samples by resampling participants within each treatment group with the number of participants fixed per group (since these totals were part of the

trial design). We resampled all participants in each arm, not just per-protocol participants, to account for the randomness in per-protocol status. We then recomputed the targeted parameter for each group using the bootstrap sample. We calculated confidence intervals from the bootstrap samples using the percentile method. Since sex and body mass index (BMI) were previously associated with T cell response magnitude (24), we considered an adjusted analysis using these covariates. A treatment group-blinded univariate analysis of sex and BMI showed a trend for an association of breadth with sex, but not BMI (data not shown). Therefore, the confidence intervals were computed with adjustment for sex; though the study groups were randomized, this adjustment can be expected to increase efficiency.

A permutation test was used for pairwise group comparisons testing the null hypothesis that the treatments had no effect. To do this we randomly permuted the treatment labels, recomputed the sex-adjusted target parameter estimates in the permuted samples and then constructed two-sided p-values by comparing the absolute value of the observed difference to the distribution of absolute differences from the permutations.

Either the mean or the median could have been used as the summary measure of central tendency for a group. Prior to our analysis we noted that the median would not be sensitive to small differences between the groups, due to the relatively small group sizes and the discrete values and small range of breadth and depth; in fact, discrete data can greatly reduce coverage probability making confidence intervals and p-values overly conservative. Though estimating the group mean for a given parameter (e.g.,

breadth) can be biased by outliers compared to a median, we specifically chose to use a non-parametric bootstrap for estimating confidence intervals and permutation tests for group comparisons, which means they are valid even when the data are not normally distributed.

All analyses are based on the intent to treat (ITT) principle including all participants in the group to which they were randomized. Summaries of responses are presented as geometric mean titers (GMT) for HIV-1 BAMA data and medians for the HIV-1 ELISPOT data. Differences in proportions were tested with two-sided Fisher's exact tests. The Kruskal-Wallis non-parametric ANOVA was used to test for differences amongst the groups. When a significant overall difference in the Kruskal-Wallis test was identified at a given time point, pair-wise tests of all possible treatment pairs were performed using the Mann-Whitney-Wilcoxon non-parametric test. The Lachenbruch test statistic was used to evaluate the composite null hypothesis of equal immune response rates between 2 groups and equal response distributions among responders in those groups (25). Two-sided 95% confidence intervals (CIs) for binomial proportions were calculated using the Wilson score test method (22). The Holm adjustment for multiple comparisons was used in testing for group differences in the binding antibody responses across multiple antigens (26). Tests with a two-sided p-value < 0.05 were considered significant.

SUPPLEMENTAL TABLES

Table S1. Participant characteristics

	C1 (N=5)	C2 (N=5)	C3 (N=5)	T1 (N=30)	T2 (N=30)	T3 (N=30)	Total (N=105)
Sex							
Male	3 (60%)	3 (60%)	2 (40%)	19 (63%)	19 (63%)	15 (50%)	61 (58%)
Female	2 (40%)	2 (40%)	3 (60%)	11 (37%)	11 (37%)	15 (50%)	44 (42%)
Ethinicity							
Hispanic or Latino/a	1 (20%)	0 (0%)	0 (0%)	3 (10%)	2 (7%)	0 (0%)	6 (6%)
Not Hispanic or Latino/a	4 (80%)	5 (100%)	5 (100%)	27 (90%)	28 (93%)	30 (100%)	99 (94%)
Race							
White	4 (80%)	4 (80%)	3 (60%)	18 (60%)	22 (73%)	18 (60%)	69 (66%)
Black/African American	0 (0%)	1 (20%)	0 (0%)	8 (27%)	5 (17%)	6 (20%)	20 (19%)
Asian	0 (0%)	0 (0%)	1 (20%)	1 (3%)	0 (0%)	4 (13%)	6 (6%)
Native Hawaiian/Pacific Islander	0 (0%)	0 (0%)	0 (0%)	0 (0%)	0 (0%)	0 (0%)	0 (0%)
Native American/Alaskan Native	0 (0%)	0 (0%)	0 (0%)	0 (0%)	0 (0%)	0 (0%)	0 (0%)
Multiracial	1 (20%)	0 (0%)	0 (0%)	2 (7%)	3 (10%)	1 (3%)	7 (7%)
Other	0 (0%)	0 (0%)	1 (20%)	1 (3%)	0 (0%)	1 (3%)	3 (3%)
Age (Years)							
Less than 18	0 (0%)	0 (0%)	0 (0%)	0 (0%)	0 (0%)	0 (0%)	0 (0%)
18 - 20	0 (0%)	0 (0%)	1 (20%)	4 (13%)	1 (3%)	6 (20%)	12 (11%)
21 - 30	2 (40%)	2 (40%)	2 (40%)	11 (37%)	11 (37%)	16 (53%)	44 (42%)
31 - 40	1 (20%)	3 (60%)	2 (40%)	7 (23%)	6 (20%)	4 (13%)	23 (22%)
41 - 50	2 (40%)	0 (0%)	0 (0%)	8 (27%)	12 (40%)	4 (13%)	26 (25%)
Over 50	0 (0%)	0 (0%)	0 (0%)	0 (0%)	0 (0%)	0 (0%)	0 (0%)
Median	40.0	31.0	22.0	30.5	33.5	26.0	30.0
Range	27-48	26-38	18-32	18-49	19-50	18-49	18-50
Vaccination Frequencies							
Day 0	5 (100%)	5 (100%)	5 (100%)	30 (100%)	30 (100%)	30 (100%)	105 (100%)
Day 28	5 (100%)	5 (100%)	5 (100%)	28 (93%)	29 (97%)	29 (97%)	101 (96%)
Day 56	5 (100%)	5 (100%)	5 (100%)	27 (90%)	29 (97%)	27 (90%)	98 (93%)
Day 112	5 (100%)	5 (100%)	5 (100%)	28 (93%)	28 (93%)	26 (87%)	97 (92%)
Day 224	5 (100%)	5 (100%)	4 (80%)	28 (93%)	25 (83%)	23 (77%)	90 (86%)

C1: Placebo for DNA Nat-B mo(0,1,2) + Placebo for MVA-CMDR mo(4,8) **C2:** Placebo for DNA CON-S mo(0,1,2) + Placebo for MVA-CMDR mo(4,8)
C3: Placebo for DNA Mosaic mo(0,1,2) + Placebo for MVA-CMDR mo(4,8)
T1: DNA Nat-B mo(0,1,2) + MVA-CMDR mo(4,8) **T2:** DNA CON-S mo(0,1,2) + MVA-CMDR mo(4,8)
T3: DNA Mosaic mo(0,1,2) + MVA-CMDR mo(4,8)
SOURCE: SCHARP (blakshmi) /trials/vaccine/p106/analysis/fsr/safety/code/run_tables_fsr.sas (SAS 9.4) 21SEP2017 11:26

Table S2. Estimates of mean breadth by group

Breadth variable	Group	Mean breadth	95% CI
Overall	Nat-B	1.13	[0.62, 1.69]
	CON-S	1.62	[0.79, 2.55]
	Mosaic	2.52	[1.19, 4.17]
Prime-matched	Nat-B	0.71	[0.36, 1.12]
	CON-S	1.18	[0.49, 2.04]
	Mosaic	2.16	[1.00, 3.52]
Heterologous	Nat-B	0.67	[0.28, 1.19]
	CON-S	1.18	[0.58, 1.90]
	Mosaic	1.50	[0.55, 2.62]
Boost-matched	Nat-B	0.74	[0.41, 1.12]
	CON-S	0.94	[0.44, 1.54]
	Mosaic	1.68	[0.84, 2.60]

Table S3. Breadth group comparisons

Breadth variable	Comparison	Mean difference	95% CI	p-value
Overall	CON-S - Nat-B	0.5	[-0.50, 1.54]	0.402
	Mosaic - Nat-B	1.39	[-0.05, 3.15]	0.017
	CON-S - Mosaic	-0.9	[-2.75, 0.75]	0.117
Prime-matched	CON-S - Nat-B	0.47	[-0.31, 1.40]	0.346
	Mosaic - Nat-B	1.45	[0.25, 2.88]	0.002
	CON-S - Mosaic	-0.98	[-2.51, 0.46]	0.048
Heterologous	CON-S - Nat-B	0.51	[-0.28, 1.32]	0.227
	Mosaic - Nat-B	0.83	[-0.24, 2.01]	0.045
	CON-S - Mosaic	-0.32	[-1.59, 0.89]	0.442
Boost-matched	CON-S - Nat-B	0.2	[-0.41, 0.87]	0.596
	Mosaic - Nat-B	0.93	[0.02, 1.91]	0.010
	CON-S - Mosaic	-0.74	[-1.81, 0.29]	0.045

SUPPLEMENTAL FIGURES

A

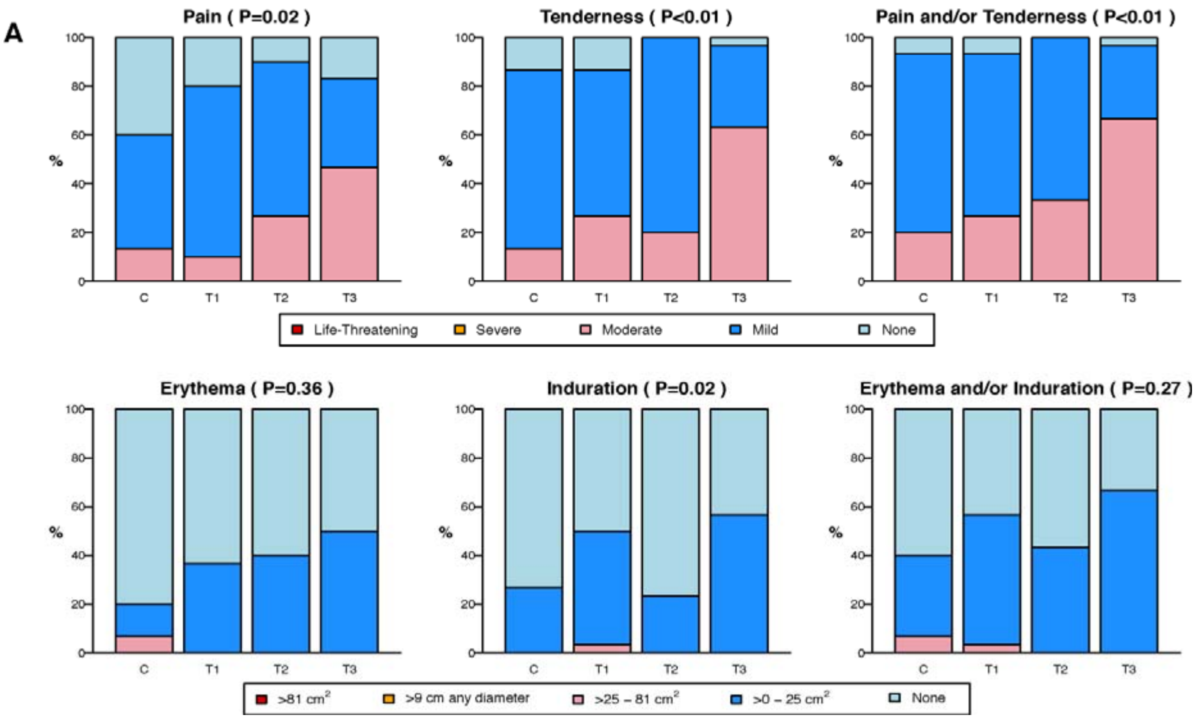

B

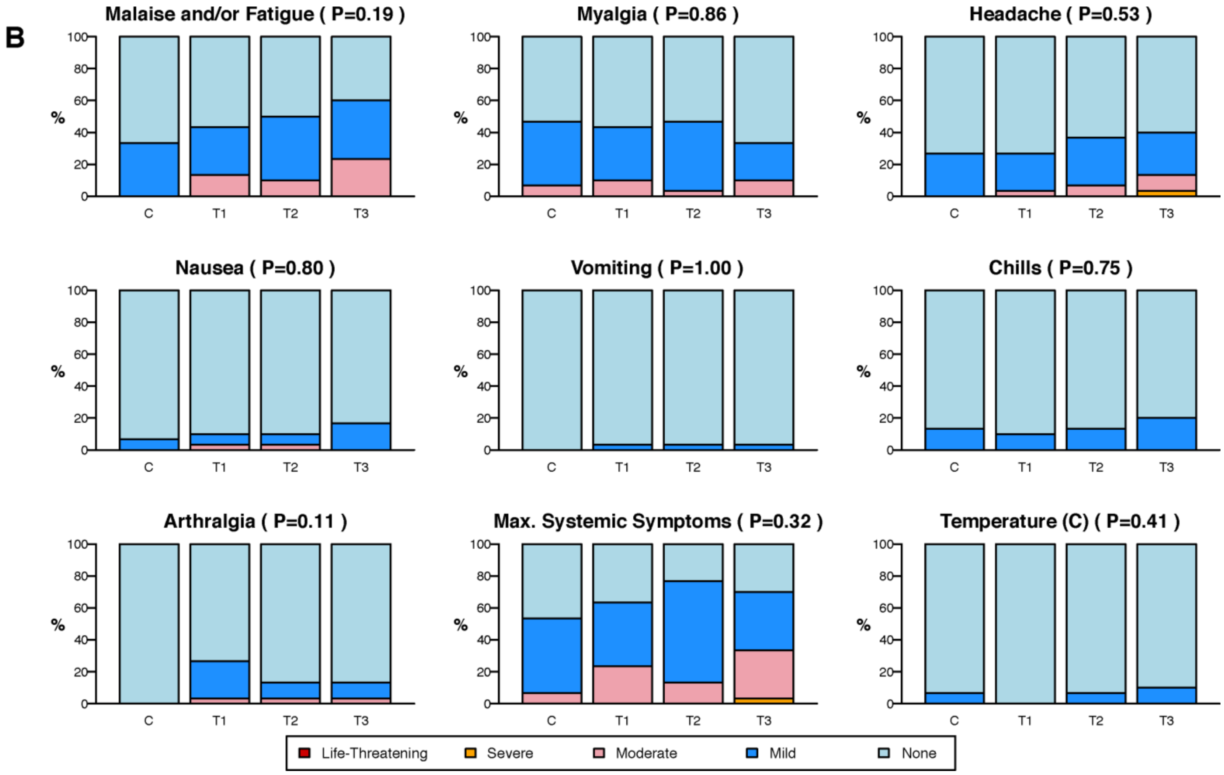

Figure S1. Local and systemic reactogenicity. Percentage of participants by study group experiencing local (A) or systemic (B) symptoms through the duration of follow-up.

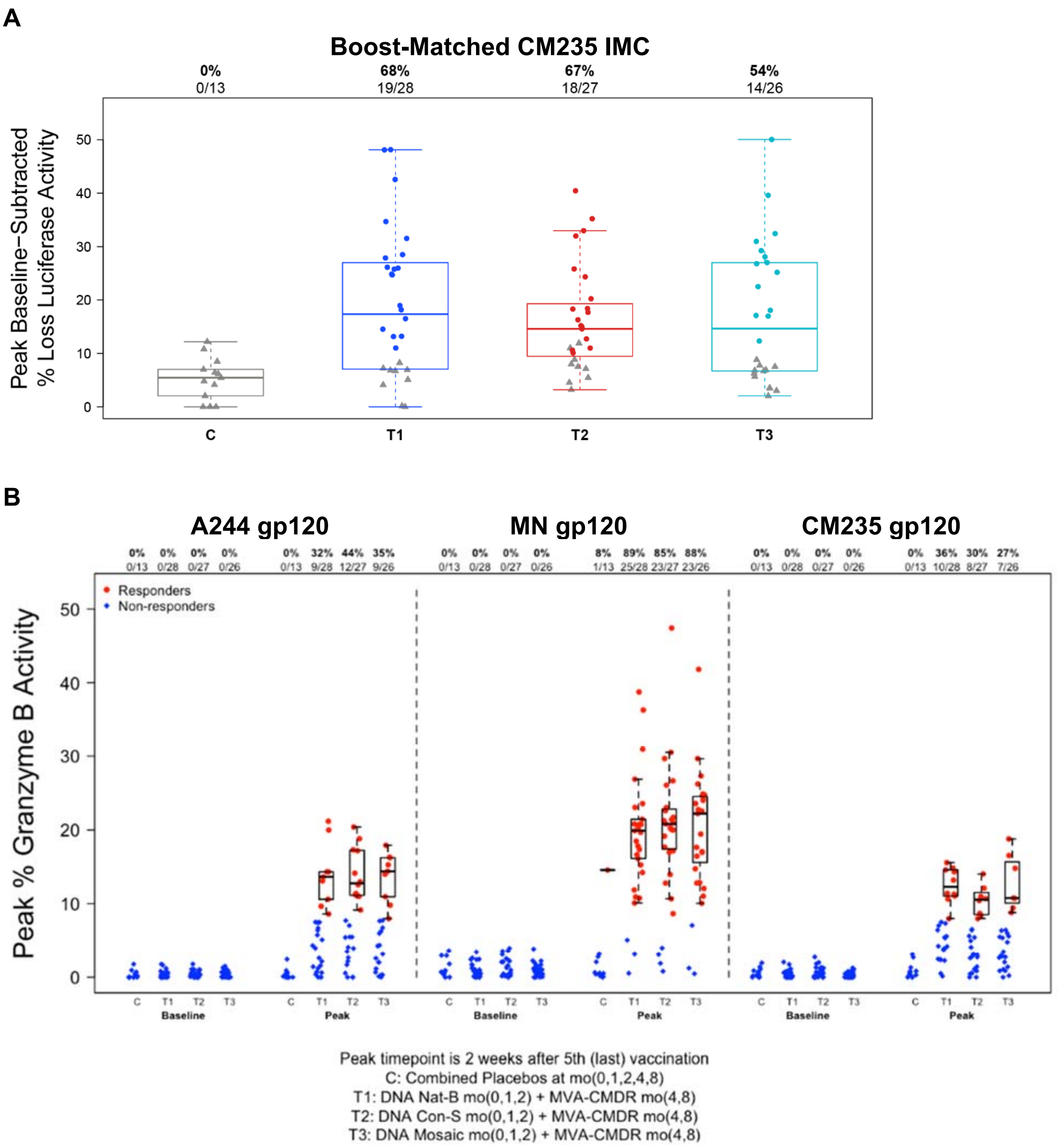

Figure S2. HIV-Env specific antibody-dependent cellular cytotoxicity (ADCC) 2 weeks post the 5th (last) vaccination. ADCC activity in sera from participants was measured by two separate methods: (A) baseline-subtracted peak inhibition of HIV infection as reduction in luciferase activity in HIV, boost-matched strain, CM235, infectious molecular clone (IMC)-infected target cells; (B) peak net Granzyme B uptake in gp120-coated target cells by GranToxiLux (GTL) staining.

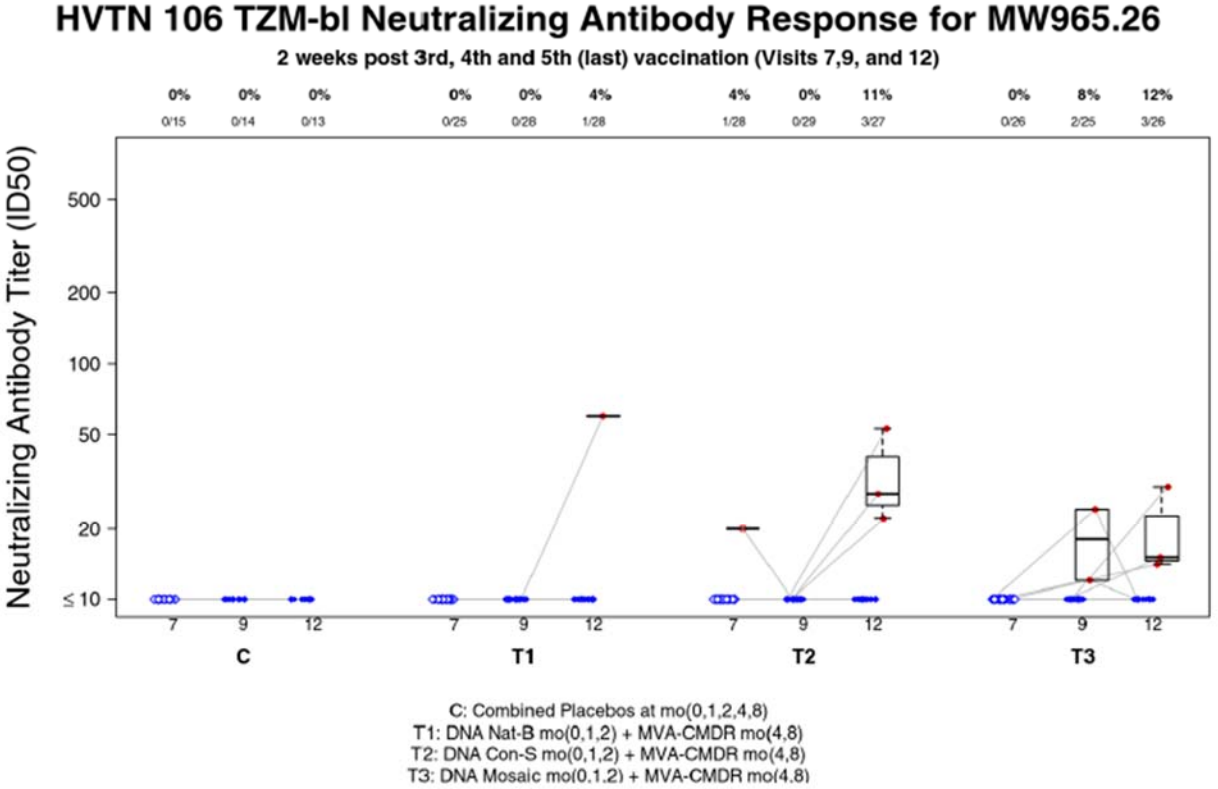

Figure S3. Neutralizing antibody responses to the Tier 1A MW965.26 HIV pseudovirus. Serum neutralizing antibody responses against HIV-1 were measured as the serum dilution resulting in a 50% reduction in Tat-regulated luciferase (Luc) reporter gene expression of the heterologous Tier 1A MW965.26 Env-pseudotyped virus in TZM-bl cells.

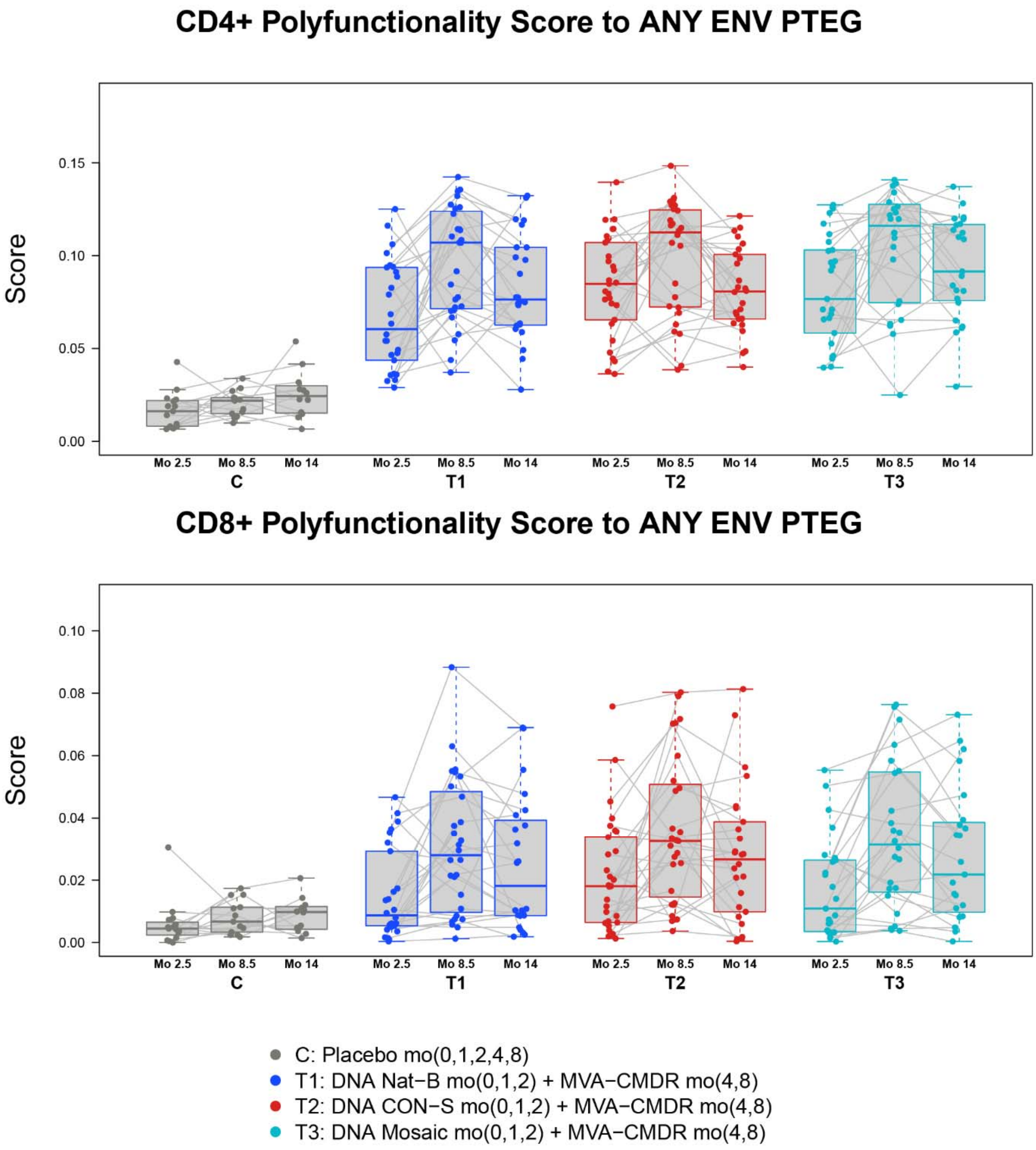

Figure S4. HIV Env-specific T-cell polyfunctionality. Analysis of (A) CD4+ and (B) CD8+ T cell responses using COMPASS (27). The polyfunctionality scores reflect both the number of HIV Env-specific cells expressing any cytokine weighted by the number of cytokines co-expressed by those cells. The cytokines evaluated included: IFN-γ, IL-2, TNF-α, CD154 and granzyme B.

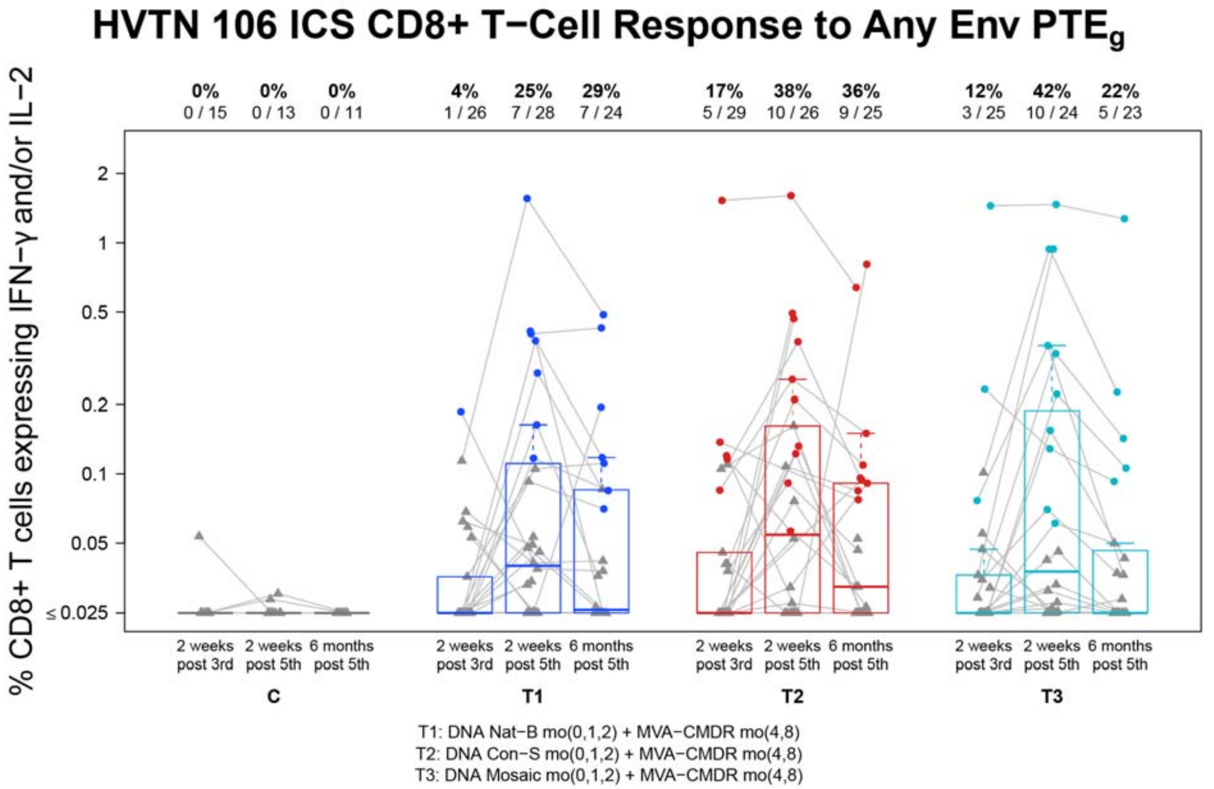

Figure S5. HIV-Env specific CD8+ T-cell responses. Frequency of HIV-1 envelope-specific CD8+ T cells was measured by intracellular cytokine staining (ICS) at 2 weeks post-3rd DNA (D70), 2 weeks post-2nd MVA (D238) and 6 months post-2nd MVA (D425, last visit) from cryopreserved peripheral blood mononuclear cells (PBMC). Responding cells expressed either IFN-γ or IL-2 in response to one of three PTE-global 15mer peptide pools; the summed frequency across these pools is displayed. Overlaid boxplots show the median and interquartile range (IQR) among responders (colored circles) and non-responders (gray triangles) in each treatment group (see Methods for ICS response call details); whiskers extend to the most extreme data points that are no more than 1.5 times the IQR. Lines connect samples from the same individual. Number and percentage of positive responses is indicated along the top of the panel for each group and timepoint.

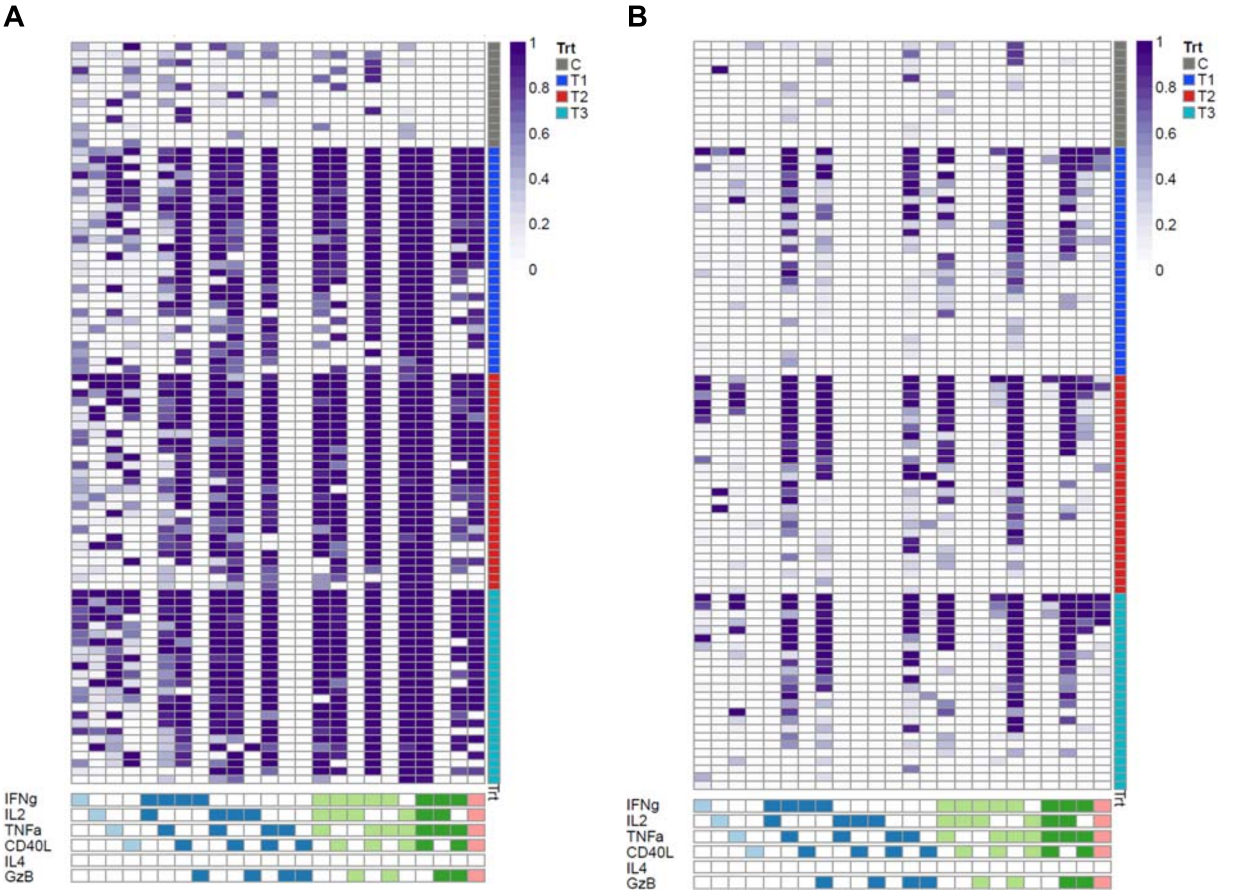

Figure S6. Cytokine expression profiles of antigen-specific T cells. Env-stimulated CD4+ T cells (A) and CD8+ T cells (B) were profiled by intracellular cytokine staining (ICS, see Methods for details); these plots represent samples collected 2 weeks post the 5th vaccination, after the MVA boost. Data were analyzed using COMPASS, which estimates the probability of response for each participant (row) and each subset of cytokine expressing T cell (each column); darker blue indicates a high probability of response. Cytokine key below each heatmap indicates the cytokines expressed by each subset, with monofunctional subsets indicated by light blue, dual functional subsets by darker blue, triple functional subsets by light green, quadruple functional subsets by dark green and the quintuple functional subset indicated by pink.

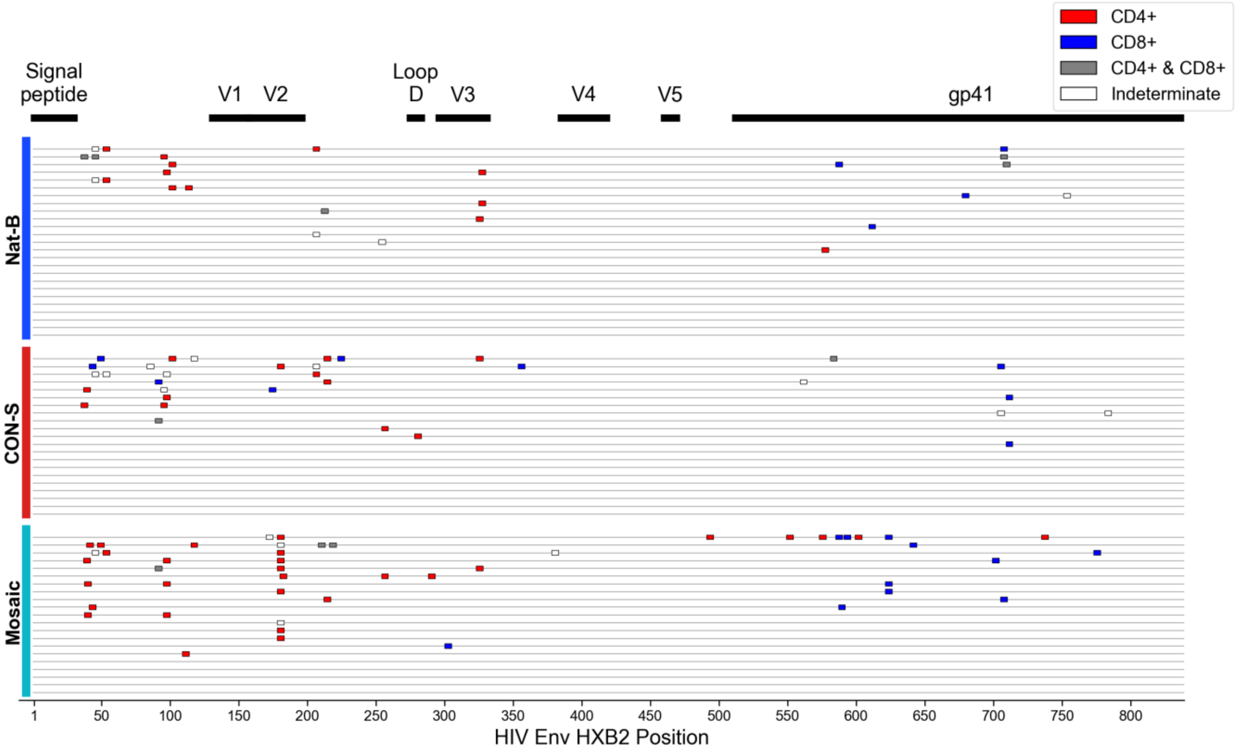

Figure S7. T cell epitope map by T cell subset. Responses to 15-mer peptides were determined from Stage 3 of IFN-γ ELISpot epitope mapping. By counting responses to overlapping peptides as a single epitope we determined the minimal set of epitopes able to explain each participant's set of peptide responses. Positive peptides were re-tested using intracellular cytokine staining (ICS) to determine CD4+/CD8+ restriction; each epitope was attributed to CD4+ T cells (red), CD8+ T cells (blue) or both (gray). Some of the epitopes could not be determined to be either CD4+ or CD8+ responses (white). Each row (y-axis tick) represents a participant (n = 80). Non-responders are included as blank rows.

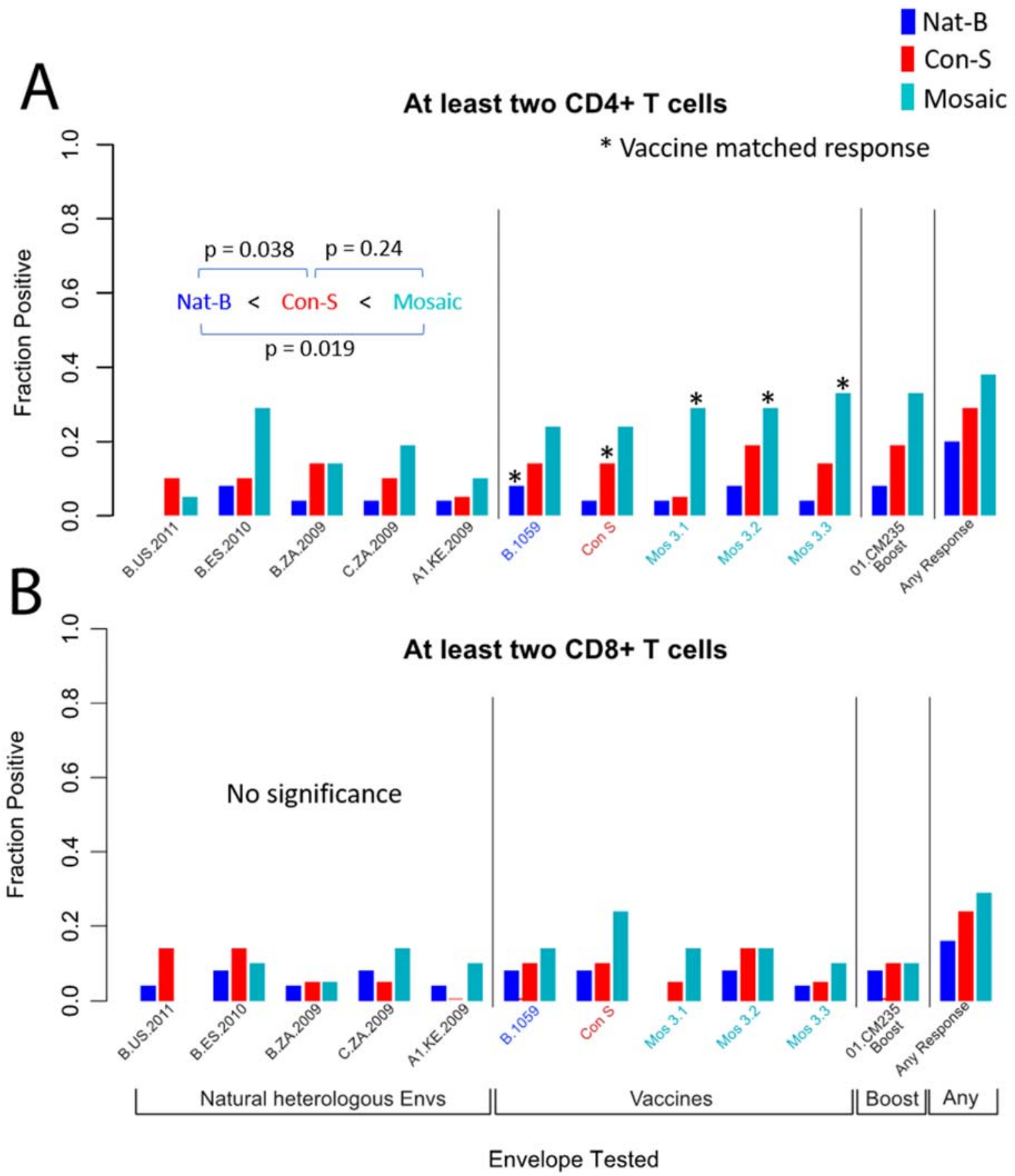

Figure S8. Fraction of individuals that made two or more detectable (A) CD4 T cells or (B) CD8 T cells. This figure is organized similarly and to Figure 5 in the main text, and illustrates the drop off in numbers of individuals that could make 2 or more CD4 or CD8 T responses against the variants tested. Very few individuals made more than one response to heterologous variants.

V2 peptide: 173-187: A highly variable epitope region

A

	Sequence	Peptide	Mosaic Responsders									
	ID		106-027	106-041	106-068	106-076	106-034	106-004	106-073	106-047	106-097	106-070
	Consensus	YALFYRLDVVPIDDN										
	M.ConS	---------------										
Prime	Mmos3.1	-----K--I--LEE-				67	70	178		207	155	545
Prime	Mmos3.2	-----K-------ND							195	240	155	325
Prime	Mmos3.3	--------I--LNEQ	113		80			132				222
	B.1059, B Nat	----N---I-----D										
	B.US.2011	R-Y-N------M---										
	B.ES.2010	R-Y-SK-------N-										
	B.ZA.2009	H----K----S-G-S										322
	C.ZA.2009	WS----P-----NE-										
	A1.KE.2009	-S------I-Q--SK										
Boost	01.CM235.MVA	H----K--I---E--		90	113	63	63	125	208	223	158	632

Peptide	Con-S	
	106-101	
YALFYRLDVVPIDDN		
---------------	88	Prime
-----K--I--LEE-	95	
-----K-------ND	72	
--------I--LNEQ	105	
----N---I-----D	85	
R-Y-N------M---		
R-Y-SK-------N-		
H----K----S-G-S		
WS----P-----NE-		
-S------I-Q--SK		
H----K--I---E--		Boost

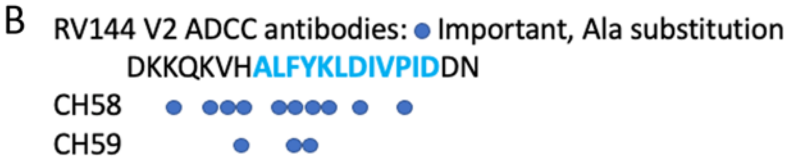

Figure S9. Responses to V2 region peptide 173-187 that overlaps with a linear B cell epitope thought to have contributed to protection in RV144. A. Positive responses in the Mos prime vaccine group (left), and Con-S vaccine group (right); there were no B-Nat group responses to this peptide. This peptide is known to be a promiscuous HLA DR binderFonseca2006 (PMID 17117012); the region highlighted in teal for mosaic responses (ALFYRLDV) is the IEDB predicted core class II binding region (see Supplemental Information Table B for details) for each of the 10 Mos responders, who carried DRB1*11, DRB1*08, or DRB1*01 class II HLAs. The region highlighted in red (FYRLDVVPI) was the IEDB predicted core epitope region for the single Con-S responder, predicted to be presented by DRB1*1001. ELISpot SFC/million response levels for each responder to each variant are shown, if a cell is shaded blue it means there was an ICS indicated a CD4 T cells response. B. This region was also targeted by ADCC B cell responses that were a correlate of protection in RV144. Two monoclonal antibodies Isolated from RV144 subjects, CH58 and CH59, bound to this region, and Ala substitutions showed critical amino acids for binding (18).

A. Vaccine antigen coverage of PTES in the M group sequences in the 6137 Env sequences in the 2019 Los Alamos database reference alignment

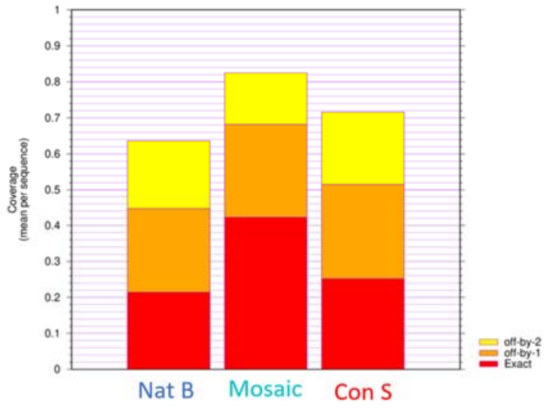

vaccine	variant count	Off-by-0	Off-by-1	Off-by-2
B.1059	6137	0.21	0.45	0.64
Mosaic	6137	0.42	0.68	0.82
ConS	6137	0.25	0.52	0.72

B. Vaccine antigen coverage of PTES in the 5 heterologous sequences used to explore the cross-reactive potential of the T cell responses in HVTN106.

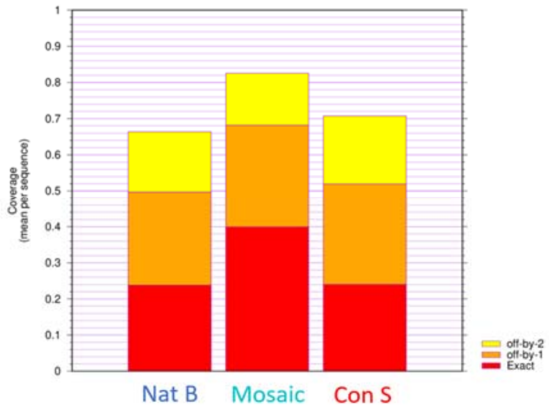

vaccine	subset count	Off-by-0	Off-by-1	Off-by-2
B.1059	5	0.24	0.50	0.66
Mosaic	5	0.40	0.68	0.83
ConS	5	0.24	0.52	0.71

Figure S10. PTE coverage of the different prime Envelopes compared to the 2019 reference alignment of M group sequences from the Los Alamos database (A), and the 5 heterologous variants to explore cross-reactive potential of T cell responses (B). This figure was made using the Epitope coverage tool at the HIV database (www.hiv.lanl.gov), to compare calculate the fraction of all linear epitope-length peptides (9-mers) in the test sequence set (the database reference alignment or the 5 heterologous proteins used in this study) that are 'covered' (i.e., matched) by some peptide in each vaccine. Results are expressed as mean PTE coverage across all test sequences.

SUPPLEMENTAL DATA

Data S1. Alignment of amino acid sequences for vaccine immunogens and heterologous sequences from which peptides were derived for epitope mapping (FASTA formatted)

```
>HXB2
MRVKEKYQHLWRWGWRWGTMLLGMLMICSATEKLWVTVYYGVPVWKEATTTLFCASDAKA
YDTEVHNVWATHACVPTDPNPQEVVLVNVTENFNMWKNDMVEQMHEDIISLWDQSLKPCV
KLTPLCVSLKCTD-----LKNDTNTNSSSGRMIMEKGEIKNCSFNISTSIRGKVQKEYAF
FYKLDIIPIDND------TTSYKLTSCNTSVITQACPKVSFEPIPIHYCAPAGFAILKCN
N-KTFNGTGPCTNVSTVQCTHGIRPVVSTQLLLNGSLAEEEVVIRSVNFTDNAKTIIVQL
NTSVEINCTRPNNNTRKRIRIQRGPGRAF-VTIGKIGNMRQAHCNISRAKWNNTLKQIAS
KLREQFGNNKTIIFKQSSGGDPEIVTHSFNCGGEFFYCNSTQLFNSTW--FNSTWSTEGS
NNTEGSDTITLPCRIKQIINMWQKVGKAMYAPPISGQIRCSSNITGLLLTRDGGNSNNE-
---SEIFRPGGGDMRDNWRSELYKYKVVKIEPLGVAPTKAKRRVVQREKRAVG-IGALFL
GFLGAAGSTMGAASMTLTVQARQLLSGIVQQQNNLLRAIEAQQHLLQLTVWGIKQLQARI
LAVERYLKDQQLLGIWGCSGKLICTTAVPWNASWS-NKSLEQIWNHTTWMEWDREINNYT
SLIHSLIEESQNQQEKNEQELLELDKWASLWNWFNITNWLWYIKLFIMIVGGLVGLRIVF
AVLSIVNRVRQGYSPLSFQTHLPTPRGPDRPEGIEEEGGERDRDRSIRLVNGSLALIWDD
LRSLCLFSYHRLRDLLLIVTRIVELLG-------RRGWEALKYWWNLLQYWSQELKNSAV
SLLNATAIAVAEGTDRVIEVVQGACRAIRHIPRRIRQGLERILL
>M.ConS
MRVRGI-QRNCQHLWRWGTLILGMLMICSAAENLWVTVYYGVPVWKEANTTLFCASDAKA
YDTEVHNVWATHACVPTDPNPQEIVLENVTENFNMWKNNMVEQMHEDIISLWDQSLKPCV
KLTPLCVTLNCTN----------VNVTNTTNNTEEKGEIKNCSFNITTEIRDKKQKVYAL
FYRLDVVPIDDN---NNNSSNYRLINCNTSAITQACPKVSFEPIPIHYCAPAGFAILKCN
D-KKFNGTGPCKNVSTVQCTHGIKPVVSTQLLLNGSLAEEEIIIRSENITNNAKTIIVQL
NESVEINCTRPNNNTRKSIRI--GPGQAFYATGDIIGDIRQAHCNISGTKWNKTLQQVAK
KLREHFNNKT-IIFKPSSGGDLEITTHSFNCRGEFFYCNTSGLFNSTW------IGNGTK
NNNNTNDTITLPCRIKQIINMWQGVGQAMYAPPIEGKITCKSNITGLLLTRDGGNNNTN-
--ETEIFRPGGGDMRDNWRSELYKYKVVKIEPLGVAPTKAKRRVVEREKRAVG-IGAVFL
GFLGAAGSTMGAASITLTVQARQLLSGIVQQQSNLLRAIEAQQHLLQLTVWGIKQLQARV
LAVERYLKDQQLLGIWGCSGKLICTTTVPWNSSWS-NKSQDEIWDNMTWMEWEREINNYT
DIIYSLIEESQNQQEKNEQELLALDKWASLWNWFDITNWLWYIKIFIMIVGGLIGLRIVF
AVLSIVNRVRQGYSPLSFQTLIPNPRGPDRPEGIEEEGGEQDRDRSIRLVNGFLALAWDD
LRSLCLFSYHRLRDFILIAARTVELLGRKG---LRRGWEALKYLWNLLQYWGQELKNSAI
SLLDTTAIAVAEGTDRVIEVVQRACRAILNIPRRIRQGLERALL
>B.1059
MRVTEI-RKNY--LWRWGIMLLGMLMICSAAEQLWVTVYYGVPVWKEATTTLFCASDAKA
YTAEAHNVWATHACVPTDPNPQEVVLENVTENFNMWKNNMVEQMHEDIISLWDQSLKPCV
KLTPLCVTLNCTDLANNTNLANNTNSSISSWEKMEKGEIKNCSFNITTVIKDKIQKNYAL
FNRLDIVPIDDDDTNVTNNASYRLISCNTSVITQACPKISFEPIPIHYCAPAGFAILKCN
D-KKFNGTGPCTNVSTVQCTHGIKPVVSTQLLLNGSLAEEEVVIRSENFTDNVKTIIVQL
NESVIINCTRPNNNTRKSITF--GPGRAFYTTGDIIGDIRKAYCNISSTQWNNTLRQIAR
RLREQFKDKT-IVFNSSSGGDPEIVMHSFNCGGEFFYCNTTQLFNSTW---NGNDTGEFN
NTGKNITYITLPCRIKQIINMWQEVGKAMYAPPIAGQIRCSSNITGILLTRDGGNSSED-
---KEIFRPEGGNMRDNWRSELYKYKVVKIEPLGVAPTKAKRRVVQREKRAVG-IGAVFL
GFLGAAGSTMGAASMTLTVQARLLLSGIVQQQNNLLRAIEAQQHLLQLTVWGIKQLQARV
LAVERYLKDQQLLGIWGCSGKLICTTAVPWNASWS-NRSLDNIWNNMTWMEWDREINNYT
NLIYNLIEESQNQQEKNEQELLELDKWASLWNWFDITKWLWYIKIFIMIVGGLVGLRIVF
VILSIVNRVRQGYSPLSFQTHLPTPRGLDRHEGTEEEGGERDRDRSGRLVDGFLTLIWID
LRSLCLFSYHRLRDLLLIVTRIVELLG-------RRGWEILKYWWNLLQYWSQELKNSAV
SLLNATAIAVAEGTDRIIEIVQRIFRAILHIPTRIRQGLERALL
>M_mos3.1
MRVKGI-RKNYQHLWRWGTMLLGMLMICSAAEQLWVTVYYGVPVWRDAETTLFCASDAKA
YEREVHNVWATHACVPTDPNPQEIVLENVTEEFNMWKNNMVDQMHEDIISLWDESLKPCV
KLTPLCVTLNCTD-------VNVTKTNSTSWGMMEKGEIKNCSFNMTTELRDKKQKVYAL
FYKLDIVPLEEN--DTISNSTYRLINCNTSAITQACPKVTFEPIPIHYCTPAGFAILKCN
D-KKFNGTGPCKNVSTVQCTHGIRPVVTTQLLLNGSLAEEEIIIRSENLTNNAKTIIVQL
NESVVINCTRPNNNTRKSIRI--GPGQTFYATGDIIGNIRQAHCNISREKWINTTRDVRK
```

KLQEHF-NKT-IIFNSSSGGDLEITTHSFNCRGEFFYCNTSKLFNSVW---GNSSNVTKV
NGTKVKETITLPCKIKQIINMWQEVGRAMYAPPIAGNITCKSNITGLLLVRDGGNVTNN-
---TEIFRPGGGNMKDNWRSELYKYKVVEIKPLGIAPTKAKRRVVEREKRAVG-LGAVFL
GFLGAAGSTMGAASMTLTVQARQLLSGIVQQQSNLLRAIEAQQHMLQLTVWGIKQLQARI
LAVERYLRDQQLLGIWGCSGKLICTTNVPWNSSWS-NKSLDEIWNNMTWMQWEKEIDNYT
SLIYTLIEESQNQQEKNEQDLLALDKWANLWNWFDISNWLWYIRIFIMIVGGLIGLRIVF
AVLSIVNRVRKGYSPLSFQTLTPNPRGPDRLGRIEEEGGEQDKDRSIRLVNGFLALAWDD
LRNLCLFSYHRLRDLLLIVTRIVELLG-------RRGWEALKYLWNLLQYWIQELKNSAV
SLLNATAIAVAEGTDRVIEVVQRACRAILHIPRRIRQGLERALL
>M_mos3.2
MRVKET-QMNWPNLWKWGTLILGLVIICSASDNLWVTVYYGVPVWKEATTTLFCASDAKA
YDTEVHNVWATYACVPTDPNPQEVVLGNVTENFNMWKNNMVEQMHEDIISLWDQSLKPCV
RLTPLCVTLNCSN--------------ANTTNTNSTEEIKNCSFNITTSIRDKVQKEYAL
FYKLDVVPIDND------NTSYRLISCNTSVITQACPKVSFEPIPIHYCAPAGFAILKCK
D-KKFNGTGPCTNVSTVQCTHGIRPVVSTQLLLNGSLAEEEVVIRSENFTNNAKTIIVHL
NKSVEINCTRPNNNTRKSIHI--GPGRAFYATGEIIGDIRQAHCNISRAKWNNTLKQIVK
KLKEQF-NKT-IIFNQSSGGDPEITTHSFNCGGEFFYCNTSGLFNSTW----NSTATQES
NNTELNGNITLPCRIKQIVNMWQEVGKAMYAPPIRGQIRCSSNITGLILTRDGGNNNST-
---NETFRPGGGDMRDNWRSELYKYKVVKIEPLGVAPTKAKRRVVQREKRAVGTIGAMFL
GFLGAAGSTMGAASLTLTVQARLLLSGIVQQQNNLLRAIEAQQHLLQLTVWGIKQLQARV
LAVERYLKDQQLLGIWGCSGKLICTTTVPWNTSWS-NKSLNEIWDNMTWMEWEREIDNYT
GLIYTLLEESQNQQEKNEQELLELDKWASLWNWFDITKWLWYIKIFIMIVGGLVGLRIVF
TVLSIVNRVRQGYSPLSFQTHLPAPRGPDRPEGIEEEGGERDRDRSGRLVDGFLAIIWVD
LRSLCLFSYHQLRDFILIAARTVELLGHSSLKGLRRGWEALKYWWNLLQYWSQELKNSAI
SLLNTTAIVVAEGTDRIIEVLQRAGRAILHIPTRIRQGLERLLL
>M_mos3.3
MRVRGI-QRNWPQWWIWGILGFWMLMICNVVGNLWVTVYYGVPVWKEAKTTLFCASDAKA
YEKEVHNVWATHACVPTDPSPQEVVLENVTENFNMWKNDMVDQMHEDVISLWDQSLKPCV
KLTHLCVTLNCTN------ATNTNYNNSTNVTSSMIGEMKNCSFNITTEIRDKSRKEYAL
FYRLDIVPLNEQ-----NSSEYRLINCNTSTITQACPKVSFDPIPIHYCAPAGYAILKCN
N-KTFNGTGPCNNVSTVQCTHGIKPVVSTQLLLNGSLAEGEIIIRSENLTDNAKTIIVHL
NESVEIVCTRPNNNTRKSVRI--GPGQAFYATGDIIGDIRQAHCNLSRTQWNNTLKQIVT
KLREQFGNKT-IVFNQSSGGDPEIVMHSFNCGGEFFYCNTTQLFNSTWENSNITQPLTLN
RTKGPNDTITLPCRIKQIINMWQGVGRAMYAPPIEGLIKCSSNITGLLLTRDGGNNSET-
-KTTETFRPGGGNMRDNWRNELYKYKVVQIEPLGVAPTRAKRRVVEREKRAVG-IGAVFL
GFLGTAGSTMGAASITLTVQARQVLSGIVQQQSNLLKAIEAQQHLLKLTVWGIKQLQTRV
LAIERYLKDQQLLGLWGCSGKLICTTAVPWNSSWS-NKSQTDIWDNMTWMQWDREISNYT
DTIYRLLEDSQNQQEKNEKDLLALDSWKNLWNWFDITNWLWYIKIFIIIVGGLIGLRIIF
AVLSIVNRCRQGYSPLSLQTLIPNPRGPDRLGGIEEEGGEQDRDRSIRLVSGFLALAWDD
LRSLCLFSYHRLRDFILIVARAVELLGRSSLRGLQRGWEALKYLGSLVQYWGLELKKSAI
SLLDTIAIAVAEGTDRIIEVIQRICRAIRNIPRRIRQGFEAALL
>01.CM235.MVA
xESKET-QMNWPNLWKWGTLILGLVIICSASDNLWVTVYYGVPVWRDADTTLFCASDAKA
HETEVHNVWATHACVPTDPNPQEIHLENVTENFNMWKNNMVEQMQEDVISLWDQSLKPCV
KLTPLCVTLNCTN---AKLTNVNNITSVSNTIGNITDEVRNCSFNMTTELRDKKQKVHAL
FYKLDIVPIEDN----KTSSEYRLINCNTSVIKQACPKISFDPIPIHYCTPAGYAILKCN
D-KNFNGTGPCKNVSSVQCTHGIKPVVSTQLLLNGSLAEEEIIIRSENLTNNAKTIIVHL
NKSVEINCTRPSNNTRTSIPI--GPGQAFYRTGDIIGDIRKAYCEINGTKWNEVLTQVTE
KLKEHFNNKT-IIFQPPSGGDLEITMHHFNCRGEFFYCNTTRLFNNTC--------IENG
TMGGCNGTIILPCKIKQIINMWQGAGQAMYAPPISGRINCVSNITGILLTRDGGAINTT-
---NETFRPGGGNIKDNWRSELYKYKVVQIEPLGIAPTRAKRRVVEREKRAVG-IGAMIF
GFLGAAGSTMGAASITLTVQARQLLSGIVQQQSNLLRAIEAQQHLLQLTVWGIKQLQARV
LAVERYLKDQKFLGLWGCSGKIICTTAVPWNSTWS-NRSYEEIWNNMTWIEWEREISNYT
NQIYEILTESQNQQDRNEKDLLELDKWASLWNWFDITKWLWYIKIFIMIIGGLLGLRIIF
AVLSIVNRVRQGYSPLSFQTPFHHQREPDRSERIEEGGGEQG------------------

>01.ClonepCM235-2.AF259954
MRVKET-QMNWPNLWKWGTLILGLVIICSASDNLWVTVYYGVPVWRDADTTLFCASDAKA
HETEVHNVWATHACVPTDPNPQEIHLENVTENFNMWKNNMVEQMQEDVISLWDQSLKPCV
KLTPLCVTLHCTN---ANLTNVNNITNVPNIIGNITDEVRNCSFNMTTELRDKKQKVHAL

FYKLDIVQIEDN---KKSSSEYRLINCNTSVIKQACPKISFDPIPIHYCTPAGYAILKCN
D-KNFNGTGPCKNVSSVQCTHGIKPVVSTQLLLNGSLAEEEIIIRSEDLTNNAKTIIVHL
NKSVEINCTRPSNNTRTSIRI--GPGQAFYRTGDIIGDIRKAYCEINGTKWNEALKQVTE
KLKEHFNNKT-IIFQPPSGGDLEITMHHFNCRGEFFYCNTTRLFNNTC--------IGNK
TMGGCNGTIILPCKIKQIINMWQGAGQAMYAPPISGRINCVSNITGILLTRDGGAINTT-
---NETFRPGGGNIKDNWRSELYKYKVVQIEPLGIAPTRAKRRVVEREKRAVG-IGAMIF
GFLGAAGSTMGAASITLTVQARQLLSGIVQQQSNLLRAIEAQQHLLQLTVWGIKQLQARV
LAVERYLKDQKFLGLWGCSGKIICTTAVPWNSTWS-NRSYEEIWNNLTWIEWEREISNYT
NQIYEILTESQDQQDRNEKDLLELDKWASLWNWFDITNWLWYIKIFIMIIGGLIGLRIIF
AVLSIVNRVRQGYSPLSFQTPSHHQREPDRPERIEEGGGEQGRDRSVRLVSGFLALAWDD
LRSLCLFSYHRLRDFILIAARTVELLGRSSLKGLRRGWEGLKYLGNLLLYWGQELKISAI
SLLDAVAIIVAGWTDRVIEVAQGAWRAILHIPRRIRQGFERALL
>B.US.2011
MRAMGI-MRNWQHLWRWGMMLLGMLMICNATDNLWVTVYYGVPVWREANTTLFCASDAKA
YETEVHNVWATHACVPTDPNPQEVKLGNVTENFNAWKNDMVEQMHEDIISLWDQSLKPCV
RLTPLCVTLNCTD----------LNATSIGSNMTLKGEIKNCTFNITTSKNDKKTTERAY
FNRLDVVPMDDN---SSSSTSYRLISCNTSVITHACPKVSFEPIPIHYCAPAGFAILKCN
D-KKFNGKGLCKNVSTVQCTHGIRPVVSTQLLLNGSLAEEEVVIRSENISNNAKTIIVHL
KESVQIICVRPNNNTRQGIHM--GPGRTFYTTGGIIGDIRQAYCNISRAEWTNTLGKIVG
KLRERF-NKT-IIFNHSSGGDLEIVTHSFNCGGEFFYCNTSALFNSTW---------NST
INTSENDTIILPCRIKQIINLWQEVGRAMYAPPIRGNISCTSNITGVLLTRDGGDDPNG-
TNDTETFRPGGGDMRDNWRNELYKYKVVKIEPLGIAPTRAKRRVVQREKRAVG-IGALFL
GFLGAAGSTMGAASVTLTVQARLLLSGIVQQQNNLLRAIEAQQHLLQLTVWGIKQLQARV
LAVERYLRDQQLLGIWGCSGKLICTTAVPWNISWS-NKSYEQIWDNMTWMQWEREIDNYT
GIIYSLIEESQIQQDKNEKDLLELDKWASLWTWFDITNWLWYIKIFIMIVGGLVGLRIVF
TVLSLVNRVRQGYSPLSFQTRLPAQRGPDRPEGIEEEGGERDRDRSRELASGFLTISWVD
LRSLCLFSYHRLRDLLLIVTRIVELLG-------RRGWEILKYWWNLLQYWSQELRNSAI
SLFNATAIAVAEGTDRIIEAIQRTVRAILHIPRRIRQGTERLLL
>B.ZA.2009
MRVEGM-RKNYQNLWRWGMMLLGILMICSA-QQLWVTVYYGVPVWKEAKTTLFCASDAKA
YDTEVHNVWATHACVPTDPNPQEIVMENVTENFNMWKNNMVEQMHEDIIELWDQSLKPCV
QLTPLCVTLNCTD-----------YGGNGTNITNSTEMKNCSFNITTSIRDKVSKEHAL
FYKLDVVSIGDS----TNNTSYRLRSCNSSVIRQACPKVSFEPIPIHYCAPAGFAILKCN
D-KKFNGTGPCTNVSTVQCTHGIRPVVSTQLLLNGSLAE-EVVIRSENFTNNAKIIIVQL
NESIEINCTRPSNNTRKSIHM--GPGGAFYTTGAVIGDIRQAHCNISGAKWERTLEQVAK
KLRKQFENKT-IVFNQSSGGDLEIVMHSFNCGGEFFYCNTTQLFNNTW---NGTTWSNNR
ATNSSKNIITLPCKIKQIINMWQQVGKAMYAPPIRGQIRCSSNITGLLLTRDGGNDNNN-
TNGSEIFRPGGGDMRDNWRSELYKYKVVTIEPVGIAPTKAKRRVVQREKRAIG-IGAVFL
GFLGAAGSTMGAASITLTVQARQLLSGIVQQQNNLLRAIEAQQHMLQLTVWGIKQLQARV
LAVERYLRDQQLLGIWGCSGKLICTTAVPWNASWS-NRSLENIWGNMTWMEWEREINNYT
GLIYNLIEESQIQQEKNEQELLELDKWASLWNWFDISNWLWYIKIFIMIVGGLIGLRIVF
TVLSIVNRVRKGYSPLSFQTHPPARREPDRPEGIEEEGGERDRGGSGQLVDGFLAIIWID
LRSLCLFSYHRLRDLLLIVTRTVELLG-------RRGWETLKYLWNLLQYWSQELKSSAV
SLFNFISIAVAEGTDRVIEVLQRAGGAVLHIPRRIRQGLERALQ
>B.ES.2010
MKVKGI-RKNCQHWWKGVMMLLGMLMICNAVQNLWVTVYYGVPVWKEATTTLFCASDAKA
YDTEAHNVWATHACVPTDPNPQEVVLGNVTENFNMWKNNMVEQMQEDIISLWDQSLKPCV
KLTPLCVTLNCTD----------VTENTVENNTINGEIKNCSFNISTEIKGKSQRERAY
FSKLDVVPIDNN-GNNANNTRYRLIHCNTSVITQACPKISFEPIPIHYCTPAGFALLKCN
D-RKFNGTGPCTNVSTVQCTHGIRPVVSTQLLLNGSLAEEEVVIRSANFTDNAKTIIVQL
KEPVKINCTRPNNNTRRGIQM--GPGRAVYATGDIIGDIRQAHCNLSKIEWEKALNQTVR
KLREQFKNKT-ISFNSSSGGDPEIVMYTFNCGGEFFYCNTTRLFNRTW----NSNDDITN
NTNSNDSDITLPCRIKQIINRWQEVGRAMYAPPIQGQIKCSSNITGMLLTRDGGHGNET-
-NNTEVFRPGGGDMRDNWRSELYKYKVVRIEPLGVAPTKAKRRVVQREKRAIG-IGAVFL
GFLGAAGSTMGAASLTLTVQARQLLSGIVQQQNNLLKAIEAQQHLLQLTVWGIKQLQARV
LAVERYLKDQQLLGIWGCSGKLICTTTVPWNDSWSNNKSLNDIWDNMTWMEWEREIDNHT
SYIYTLIEKAQNQQEKNEQELLELDQWASLWNWFSISNWLWYIRIFIIIVGGLVGLRIVM
AVLSVVNRVRQGYSPLSLQTHLPAQRGPDRPDGIEEEGGEKGRGRSGQLVKGFSALFWDD
LRSLCLFSYHRLTDLLLIAARIVGLLG-------RRGWEILKYWWNLLQYWGQELKDSAV
SLLNATAIAVAEGTDRVIEIIQRAGRAIRHIPTRIRQGLERALL
>A1.KE.2009

```
MRVMGI-QMNYQHLLTWGTIILGMILICSPAENLWVTVYYGVPVWREADTTLFCASDAKA
YETEKHNVWATHACVPTDPNPQEIHLANVTENFDMWKNNMVEQMHTDIISLWDQSLQPCV
KLTPLCVTMNCSE----PKFNNTNFNSTGSNSTGIQEEMRNCSFNMTTELKDKKKEVYSL
FYRLDIVQIDSK---KGNSSDYRLINCNTSAVKQACPKVSFNPIPIHYCAPAGFAILKCR
D-EDFNGTGPCKNVSTVQCTHGIMPVVSTQLLLNGSLAKENVQIRSENISNNAKIILVQL
AHPVRINCTRPGNNTRKSIHM--GPGQAFYARGDVIGDIRQAYCNVSSSEWSNTLYKVAE
QLRKHYGNETTIKFTNHSGGDLEVTTHSFNCGGEFFYCNTTNLFNSSI---PFNASERAN
NTNSTDDIITLQCRIKQIVRMWQRVGQAMYAPPIPGVIRCESNITGLMLTWDGGSKNITE
GNRTETFRPGGGDMRDNWRSELYKYKVVKIEPLGVAPTKARRRVVGREKRAVG-IGAVFL
GFLGAAGSTMGAASVTLTVQARQLLSGIVQQQSNLLRAIEAQQQLLKLTVWGIKQLQARV
LALERYLKDQQLLGIWGCSGRLICTTNVPWNSSWS-NKSYNEIWDNMTWLQWDREIENYT
QIIYGLIEESQNQQEKNEQDLLSLNKWADLWSWFNITNWLWYIKIFIMIVGGLIGLRIVF
AVLSVINRVRQGYSPLSFQTHLPNPGGLDRPERIEEEDGEQGRTRSIRLVSGFLALAWDD
LRSLCLFSYHRLRDFILIAARTVELLGHSSLKGLRLGWEGLKYLWNLLVYWGRELKTSAI
SLVDTIAIVVAGWTDRAIEIGQRIGRAILHIPIRIRQGLERALL
>C.ZA.2009
MRVKGI-LRNCPQWWIWGILGLWMLLICNG-ENSWVTVYYGVPVWKEAKTTLFCASDAKA
YEKEVHNVWATHACVPTDPNPQEIFMENVTENFNMWKNDMVDQMHEDIISLWDQSLKPCV
KLTPLCVTLNCTPCVNQTCSNSTVNSTVTPINSTGNEQMTNCSFNVTTEIRDKEKKAWSL
FYRPDVVPINEN------SSEYILINCNSSTITQACPKVTFDPIPIHYCAPAGYAILKCN
DNKTFNGTGPCKNVSTVQCTHGIKPVVSTQFLLNGSLAVEDIIIRSENLTDNIKTIIVHL
NESVEINCTRPSNNTRKSMRI--GPGQIFYAYGDIIGDIRQAYCNISQSQWNKTLQRVRE
KLKEHFPNKT-INFQPSSGGDLEITTHSFNCRGEFFYCNTTRLFNNTE--------SNST
ITGNHSDTITLPCRIKQFINMWQEVGRAMYAPPIAGTITCISNITGLLLVRDGGIPNEN-
---TEIFRPQGGNMKDNWRSELYKYKVVEIKPLGIAPTTAKRRVVQREKRAVG-IGAVLL
GFLGAAGSTMGAASITLTVQARHLLSGIVQQQSNLLRAIEAQQHMLQLTVWGIKQLQTRV
LAIERYLKDQQLLGLWGCSGKLICTTAVPWNSSWS-NKSQIDIWENMTWMQWDKEISNYT
YTIYKLLEDSQSQQEQNEKDLLALDSWNNLWSWFSITNWLWYIKLFIMIVGGLIGLRIIF
AVLSIVNRVRQGYSPLSFQTLTPSPREPDRLGRIEEEGGEQDRNRSIRLVSGFLALAWDD
LRSLCLFSYHRLKDFILVTARAVELLGRSSLRGLQRGWEILKYLGSLVQYWCLELKKSAI
SLFDTIAIRVAEGTDSIIIVTQRIFRAILNIPTRIRQGLEAALL
```

SUPPLEMENTAL REFERENCES

1. Catanzaro, A.T., Koup, R.A., Roederer, M., Bailer, R.T., Enama, M.E., Moodie, Z., Gu, L., Martin, J.E., Novik, L., Chakrabarti, B.K., et al. 2006. Phase 1 safety and immunogenicity evaluation of a multiclade HIV-1 candidate vaccine delivered by a replication-defective recombinant adenovirus vector. *J Infect Dis* 194:1638-1649.
2. Churchyard, G.J., Morgan, C., Adams, E., Hural, J., Graham, B.S., Moodie, Z., Grove, D., Gray, G., Bekker, L.G., McElrath, M.J., et al. 2011. A phase IIA randomized clinical trial of a multiclade HIV-1 DNA prime followed by a multiclade rAd5 HIV-1 vaccine boost in healthy adults (HVTN204). *PLoS One* 6:e21225.
3. Hulot, S.L., Korber, B., Giorgi, E.E., Vandergrift, N., Saunders, K.O., Balachandran, H., Mach, L.V., Lifton, M.A., Pantaleo, G., Tartaglia, J., et al. 2015. Comparison of Immunogenicity in Rhesus Macaques of Transmitted-Founder, HIV-1 Group M Consensus, and Trivalent Mosaic Envelope Vaccines Formulated as a DNA Prime, NYVAC, and Envelope Protein Boost. *J Virol* 89:6462-6480.
4. Liao, H.X., Sutherland, L.L., Xia, S.M., Brock, M.E., Scearce, R.M., Vanleeuwen, S., Alam, S.M., McAdams, M., Weaver, E.A., Camacho, Z., et al. 2006. A group M consensus envelope glycoprotein induces antibodies that neutralize subsets of subtype B and C HIV-1 primary viruses. *Virology* 353:268-282.
5. Weaver, E.A., Camacho, Z.T., and Gao, F. 2010. Similar T-cell immune responses induced by group M consensus env immunogens with wild-type or minimum consensus variable regions. *AIDS Res Hum Retroviruses* 26:577-584.

6. Weaver, E.A., Lu, Z., Camacho, Z.T., Moukdar, F., Liao, H.X., Ma, B.J., Muldoon, M., Theiler, J., Nabel, G.J., Letvin, N.L., et al. 2006. Cross-subtype T-cell immune responses induced by a human immunodeficiency virus type 1 group m consensus env immunogen. *J Virol* 80:6745-6756.
7. Santra, S., Liao, H.X., Zhang, R., Muldoon, M., Watson, S., Fischer, W., Theiler, J., Szinger, J., Balachandran, H., Buzby, A., et al. 2010. Mosaic vaccines elicit CD8+ T lymphocyte responses that confer enhanced immune coverage of diverse HIV strains in monkeys. *Nat Med* 16:324-328.
8. Santra, S., Muldoon, M., Watson, S., Buzby, A., Balachandran, H., Carlson, K.R., Mach, L., Kong, W.P., McKee, K., Yang, Z.Y., et al. 2012. Breadth of cellular and humoral immune responses elicited in rhesus monkeys by multi-valent mosaic and consensus immunogens. *Virology* 428:121-127.
9. Earl, P.L., Cotter, C., Moss, B., VanCott, T., Currier, J., Eller, L.A., McCutchan, F., Birx, D.L., Michael, N.L., Marovich, M.A., et al. 2009. Design and evaluation of multi-gene, multi-clade HIV-1 MVA vaccines. *Vaccine* 27:5885-5895.
10. Currier, J.R., Ngauy, V., de Souza, M.S., Ratto-Kim, S., Cox, J.H., Polonis, V.R., Earl, P., Moss, B., Peel, S., Slike, B., et al. 2010. Phase I safety and immunogenicity evaluation of MVA-CMDR, a multigenic, recombinant modified vaccinia Ankara-HIV-1 vaccine candidate. *PLoS One* 5:e13983.
11. Sandstrom, E., Nilsson, C., Hejdeman, B., Brave, A., Bratt, G., Robb, M., Cox, J., Vancott, T., Marovich, M., Stout, R., et al. 2008. Broad immunogenicity of a multigene, multiclade HIV-1 DNA vaccine boosted with heterologous HIV-1 recombinant modified vaccinia virus Ankara. *J Infect Dis* 198:1482-1490.
12. Walsh, S.R., Wilck, M.B., Dominguez, D.J., Zablowsky, E., Bajimaya, S., Gagne, L.S., Verrill, K.A., Kleinjan, J.A., Patel, A., Zhang, Y., et al. 2013. Safety and immunogenicity of modified vaccinia Ankara in hematopoietic stem cell transplant recipients: a randomized, controlled trial. *J Infect Dis* 207:1888-1897.
13. Guardo, A.C., Gomez, C.E., Diaz-Brito, V., Pich, J., Arnaiz, J.A., Perdiguero, B., Garcia-Arriaza, J., Gonzalez, N., Sorzano, C.O.S., Jimenez, L., et al. 2017. Safety and vaccine-induced HIV-1 immune responses in healthy volunteers following a late MVA-B boost 4 years after the last immunization. *PLoS One* 12:e0186602.
14. Joachim, A., Munseri, P.J., Nilsson, C., Bakari, M., Aboud, S., Lyamuya, E.F., Tecleab, T., Liakina, V., Scarlatti, G., Robb, M.L., et al. 2017. Three-Year Durability of Immune Responses Induced by HIV-DNA and HIV-Modified Vaccinia Virus Ankara and Effect of a Late HIV-Modified Vaccinia Virus Ankara Boost in Tanzanian Volunteers. *AIDS Res Hum Retroviruses* 33:880-888.
15. Andrasik, M.P., Karuna, S.T., Nebergall, M., Koblin, B.A., Kublin, J.G., and Network, N.H.V.T. 2013. Behavioral risk assessment in HIV Vaccine Trials Network (HVTN) clinical trials: a qualitative study exploring HVTN staff perspectives. *Vaccine* 31:4398-4405.
16. Campion, S.L., Brenna, E., Thomson, E., Fischer, W., Ladell, K., McLaren, J.E., Price, D.A., Frahm, N., McElrath, J.M., Cohen, K.W., et al. 2021. Preexisting memory CD4+ T cells contribute to the primary response in an HIV-1 vaccine trial. *J Clin Invest* 131.
17. Pollara, J., Hart, L., Brewer, F., Pickeral, J., Packard, B.Z., Hoxie, J.A., Komoriya, A., Ochsenbauer, C., Kappes, J.C., Roederer, M., et al. 2011. High-throughput quantitative analysis of HIV-1 and SIV-specific ADCC-mediating antibody responses. *Cytometry A* 79:603-612.
18. Liao, H.X., Bonsignori, M., Alam, S.M., McLellan, J.S., Tomaras, G.D., Moody, M.A., Kozink, D.M., Hwang, K.K., Chen, X., Tsao, C.Y., et al. 2013. Vaccine induction of antibodies against a structurally heterogeneous site of immune pressure within HIV-1 envelope protein variable regions 1 and 2. *Immunity* 38:176-186.
19. Pollara, J., Bonsignori, M., Moody, M.A., Liu, P., Alam, S.M., Hwang, K.K., Gurley, T.C., Kozink, D.M., Armand, L.C., Marshall, D.J., et al. 2014. HIV-1 vaccine-induced C1 and V2 Env-specific antibodies synergize for increased antiviral activities. *J Virol* 88:7715-7726.
20. Moncunill, G., Dobaño, C., McElrath, M.J., and De Rosa, S.C. 2015. OMIP-025: evaluation of human T- and NK-cell responses including memory and follicular helper phenotype by intracellular cytokine staining. *Cytometry A* 87:289-292.
21. Li, F., Malhotra, U., Gilbert, P.B., Hawkins, N.R., Duerr, A.C., McElrath, J.M., Corey, L., and Self, S.G. 2006. Peptide selection for human immunodeficiency virus type 1 CTL-based vaccine evaluation. *Vaccine* 24:6893-6904.

22. Agresti, A., and Coull, B.A. 1998. Approximate Is better than "exact" for interval estimation of binomial proportions. *Am Stat* 52:119-126.
23. Fiore-Gartland, A., Manso, B.A., Friedrich, D.P., Gabriel, E.E., Finak, G., Moodie, Z., Hertz, T., De Rosa, S.C., Frahm, N., Gilbert, P.B., et al. 2016. Pooled-Peptide Epitope Mapping Strategies Are Efficient and Highly Sensitive: An Evaluation of Methods for Identifying Human T Cell Epitope Specificities in Large-Scale HIV Vaccine Efficacy Trials. *PLoS One* 11:e0147812.
24. Hopkins, K.L., Laher, F., Otwombe, K., Churchyard, G., Bekker, L.G., DeRosa, S., Nchabeleng, M., Mlisana, K., Kublin, J., and Gray, G. 2014. Predictors of HVTN 503 MRK-AD5 HIV-1 gag/pol/nef vaccine induced immune responses. *PLoS One* 9:e103446.
25. Lachenbruch, P.A. 2001. Comparisons of two-part models with competitors. *Stat Med* 20:1215-1234.
26. Holm, S. 1979. A simple sequentially rejective multiple test procedure. *Scandinavian Journal of Statistics* 6:65-70.
27. Lin, L., Finak, G., Ushey, K., Seshadri, C., Hawn, T.R., Frahm, N., Scriba, T.J., Mahomed, H., Hanekom, W., Bart, P.A., et al. 2015. COMPASS identifies T-cell subsets correlated with clinical outcomes. *Nat Biotechnol* 33:610-616.
